# Supplementary material for: Gene-environment interactions explain a substantial portion of variability of common neuropsychiatric disorders
Source: Cell Rep Med. 2022 Sep 6;3(9):100736. doi: 10.1016/j.xcrm.2022.100736 (PMC9512674; doi:10.1016/j.xcrm.2022.100736)
Supplement: Document S1. Figures S1 and S2 and Table S1 [file mmc1.pdf]

Cell Reports Medicine, Volume *3*

Supplemental information

Gene-environment interactions explain a substantial portion of variability of common neuropsychiatric disorders

Hanxin Zhang, Atif Khan, and Andrey Rzhetsky

Gene-Environment Interactions Explain a Substantial Portion of Variability of Common Neuropsychiatric Disorders: Supplementary Figures and Table

Hanxin Zhang[1, 2], Atif Khan[2], and Andrey Rzhetsky[1, 2, 3, 4 *]

[1]Committee on Genetics, Genomics and Systems Biology, The University of Chicago, Chicago, IL, 60637, US.

[2]Department of Medicine, and Institute of Genomics and Systems Biology, The University of Chicago, Chicago, IL, 60637, US.

[3]Department of Human Genetics and Committee on Quantitative Methods in Social, Behavioral, and Health Sciences, The University of Chicago, Chicago, IL, 60637, US.

[4]Lead contact

[*]Correspondence to: andrey.rzhetsky@uchicago.edu

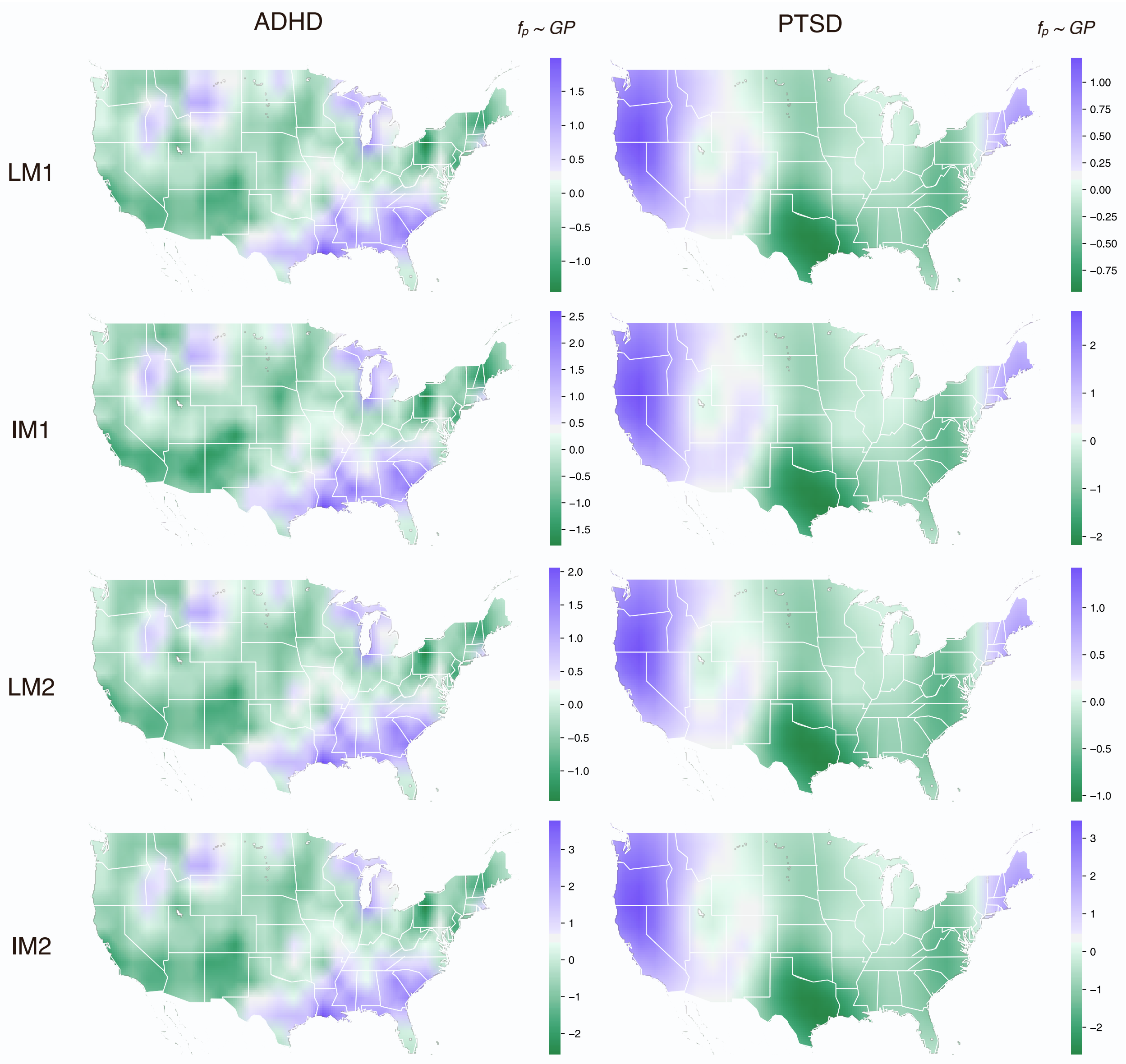

Figure S1. The mean estimates of the geographic random effects for ADHD and PTSD, related to Figure 1.

These figure plots show the posterior mean estimates of the geographic random effects for ADHD and PTSD ($f_p(\mathbf{x})$ (see Expression (8) in the *Models* section of the *Supplementary Information*). We modeled the geographic random effects $f_p(\mathbf{x})$ using a Gaussian process assuming that adjacent geographical locations have close-value random effects (assumption of smoothness). For both ADHD and PTSD, all models across the complexity spectrum (LM1, IM1, LM2, and IM2; note that LM0 does not incorporate the geographic random effect term) give nearly identical patterns of $f_p(\mathbf{x})$ estimates across the continental US. We did include residents of Hawaii and Alaska in our estimation process, but the results are not shown here. We omitted these results because the discontinuity between the geographic locations disobeys the Gaussian process's presumptions. The Gaussian process's poor extrapolation power also makes it difficult to estimate the random effects related to outlying states such as Alaska and Hawaii. Our data does not record any residents of other non-continental US islands.

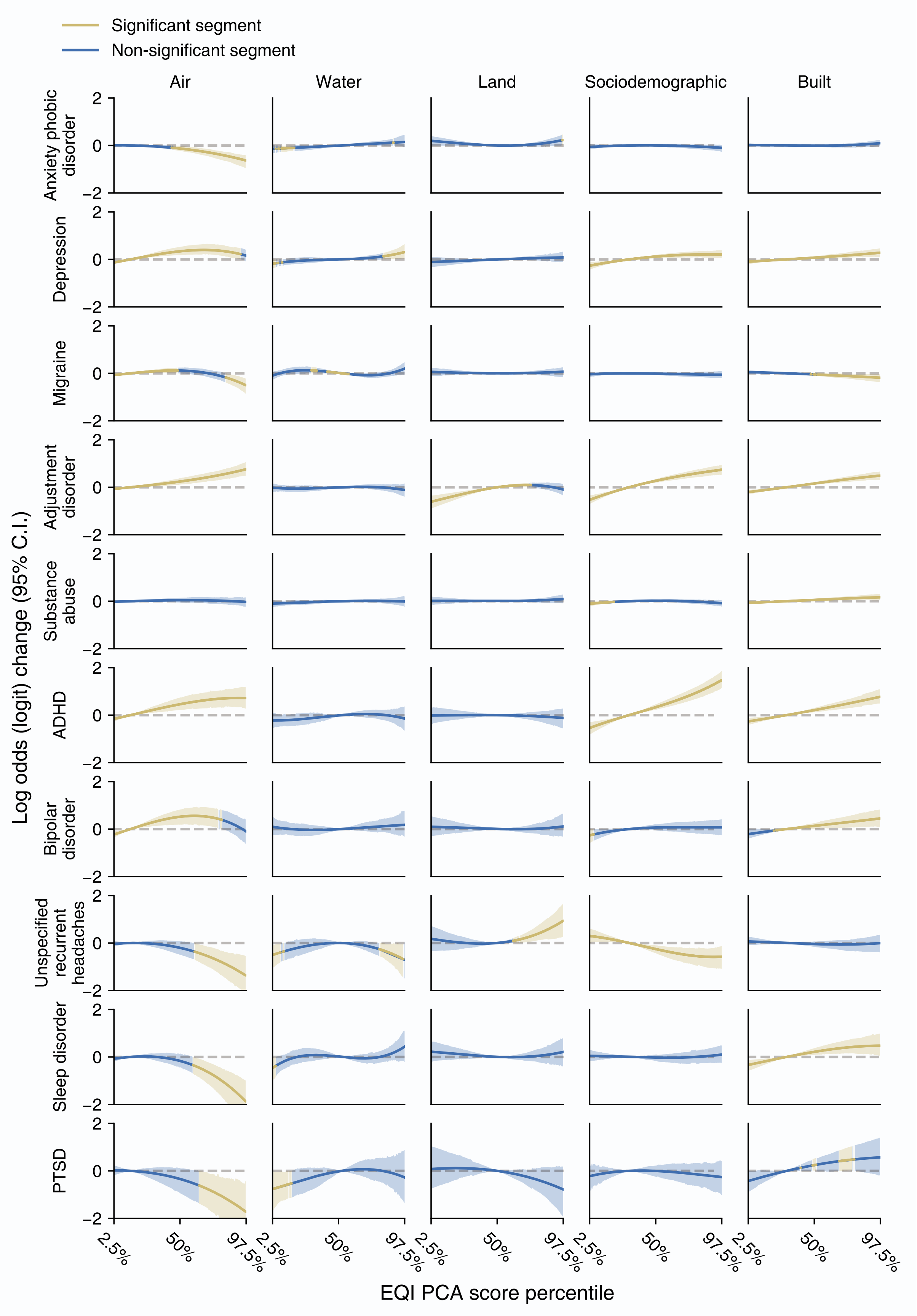
Significant segment
Non-significant segment
Air
Water
Land
Sociodemographic
Built
Anxiety phobic disorder
Depression
Migraine
Adjustment disorder
Substance abuse
ADHD
Bipolar disorder
Unspecified recurrent headaches
Sleep disorder
PTSD
Log odds (logit) change (95% C.I.)
2
0
−2
2.5%
50%
97.5%
EQI PCA score percentile

Figure S2. The nonlinear effects estimated for the five EQI domains (air, water, land, sociodemographic, and built environment) given by each disease's WAIC-best model, related to Figure 2.

Each EQI domain contains multiple relevant variables. We employed a PCA procedure to summarize and standardize domain-specific variables into one-dimensional PCA scores. According to the publisher (EPA), higher scores generally reflect worse environmental quality. We converted these PCA scores into percentiles based on the score distribution covering the whole population. We used the 2.5th to 97.5th percentiles as the x-axis limits of the plots. In the line plots, the blue segments indicate EQI regions that do not change the log odds significantly (statistically equal to zero, 95 percent credible intervals). The olive segments represent those EQI regions that significantly alter the disease log odds (statistically non-zero regions, 95 percent credible intervals).

Supplementary Information Tables

Table S1. The simulation result of the five experiment sets (see Section *Statistical Simulations of Inference Under the Assumed Model* in the *STAR Methods*).

**Statistics**	h^2	e^2	he^2
**Experiment set 1:** $\sigma_G{}^2 = 1, \sigma_E{}^2 = 5$			
**Coverage probability estimate**	0.98	0.78	0.92
**Experiment set 2:** $\sigma_G{}^2 = 2, \sigma_E{}^2 = 4$			
**Coverage probability estimate**	0.90	0.86	0.88
**Experiment set 3:** $\sigma_G{}^2 = 3, \sigma_E{}^2 = 3$			
**Coverage probability estimate**	0.89	0.97	0.94
**Experiment set** 4: $\sigma_G{}^2 = 4, \sigma_E{}^2 = 2$			
**Coverage probability estimate**	0.86	0.98	0.93
**Experiment set 5:** $\sigma_G{}^2 = 5, \sigma_E{}^2 = 1$			
**Coverage probability estimate**	0.83	0.89	0.89

Average coverage probability estimate = 0.90
