# Supplementary material for: Gene-environment interactions explain a substantial portion of variability of common neuropsychiatric disorders
Source: Cell Rep Med. 2022 Sep 6;3(9):100736. doi: 10.1016/j.xcrm.2022.100736 (PMC9512674; doi:10.1016/j.xcrm.2022.100736)
Supplement: Document S2. Article plus supplemental information [file mmc2.pdf]

Cell Reports Medicine

Article

Gene-environment interactions explain a substantial portion of variability of common neuropsychiatric disorders

Graphical abstract

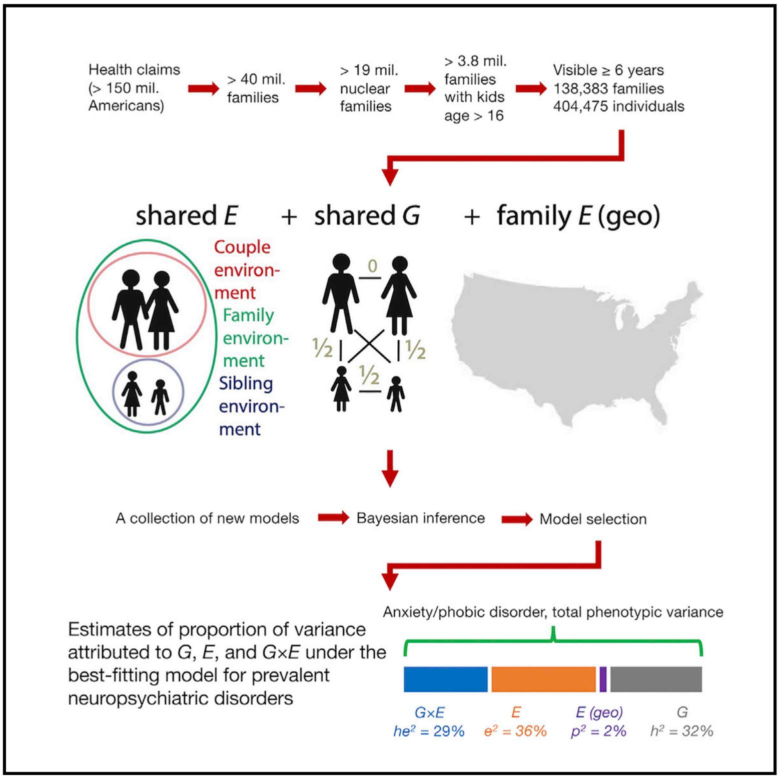

Highlights

- G-by-E interactions contribute to the etiology of neuropsychiatric disorders
- Environmental quality is associated with the risk of neuropsychiatric disorders
- Geographic locations explain a part of variability of neuropsychiatric disorders
- Identifying specific G-by-E interactions might be the future of personalized medicine

Authors

Hanxin Zhang, Atif Khan, Andrey Rzhetsky

Correspondence

andrey.rzhetsky@uchicago.edu

In brief

Zhang et al. show that gene-environment interactions account for a significant portion of the phenotypic variance of common neuropsychiatric disorders: over 20% for ADHD, migraine, and anxiety/phobic disorder and close to 30% for recurrent headaches, sleep disorders, and post-traumatic stress disorder.

Zhang et al., 2022, Cell Reports Medicine *3*, 100736
September 20, 2022 © 2022 The Author(s).
https://doi.org/10.1016/j.xcrm.2022.100736

CellPress

Cell Reports Medicine

CellPress
OPEN ACCESS

Article

Gene-environment interactions explain a substantial portion of variability of common neuropsychiatric disorders

Hanxin Zhang,[1,2] Atif Khan,[2] and Andrey Rzhetsky[1,2,3,4,*]
[1]Committee on Genetics, Genomics and Systems Biology, The University of Chicago, Chicago, IL 60637, USA
[2]Department of Medicine, Institute of Genomics and Systems Biology, The University of Chicago, Chicago, IL 60637, USA
[3]Department of Human Genetics and Committee on Quantitative Methods in Social, Behavioral, and Health Sciences, The University of Chicago, Chicago, IL 60637, USA
[4]Lead contact
*Correspondence: andrey.rzhetsky@uchicago.edu
https://doi.org/10.1016/j.xcrm.2022.100736

SUMMARY

In complex diseases, the phenotypic variability can be explained by genetic variation (G), environmental stimuli (E), and interaction of genetic and environmental factors (G-by-E effects), among which the contribution G-by-E remains largely unknown. In this study, we focus on ten major neuropsychiatric disorders using data for 138,383 United States families with 404,475 unique individuals. We show that, while gene-environment interactions account for only a small portion of the total phenotypic variance for a subset of disorders (depression, adjustment disorder, substance abuse), they explain a rather large portion of the phenotypic variation of the remaining disorders: over 20% for migraine and close to or over 30% for anxiety/phobic disorder, attention-deficit/hyperactivity disorder, recurrent headaches, sleep disorders, and post-traumatic stress disorder. In this study, we have incorporated—in the same analysis—clinical data, family pedigrees, the spatial distribution of individuals, their socioeconomic and demographic confounders, and a collection of environmental measurements.

INTRODUCTION

The study of phenotypic trait heritability is one of the most important topics in biology. Heritability is the proportion of the overall trait variance that can be explained by genetic variation under an explicitly defined genetic penetrance model. In practice, heritability has been used to indicate the strength of a trait's response to artificial selection in domesticated organisms. As expected, this is not very productive when attempting to artificially select for traits with low heritability. Since the earliest days in the field, geneticists have imagined the possibility of the existence of complex interactions between genetic variation and environmental stimuli, often denoted as $G \times E$ ("the G-by-E effect"). However, measuring such interactions in real-world data has proved rather difficult.[1–6] A hypothetically ideal dataset for analyzing how environmental and genetic factors affect human disease would possess the following properties:

(1) The data would need to contain the complete germline genomic sequence for a large number of individuals. While such human datasets are available today, our ideal data would link all study individuals into a unified genetic pedigree, which would allow for the inference of genetic models associated with the vertical trait transmission.
(2) For each study participant, the data would contain complete phenotypic profiles across all human traits. This is very challenging, as myriads of phenotypic characteristics, which change throughout individuals' lives, would have to be recorded. Currently—at best—human observational data contains snapshots of medical histories and collections of biometric, metabolic, and clinical measurements for each individual, for a limited window of their ontogenetic timeline, usually capturing a few years (or less) of each timeline.
(3) For each individual, the data would document a complete record of exterior environmental events and interventions, including exposures to changes in climate, tidal waves of uncountable bacterial, viral, fungal, and protozoic encounters, billions of environmental molecular species entering the individual's body over time, and exposure to electromagnetic fields and the bombardment of elementary particles, as well as the individual's diet, exercise routines, and social support structure. These factors are typically the worst-documented part of human life, because the set of relevant stimuli is astronomically vast and must be recorded continuously from conception to death.

Were we able to generate a gigantic *genotype* × *environment* × *phenotype* × *time* data matrix, it would be used to fit a battery of increasingly complex mathematical models encapsulating the probability of observing a specific phenotype, given genetic and environmental inputs. The models that we are comparing

© 2022 The Author(s).
This is an open access article under the CC BY license (http://creativecommons.org/licenses/by/4.0/).

represent alternative theories of how genetics, environments, and their interactions contribute to disease onset. The degree of each model's fit to the data for a disease then explicitly quantifies the corresponding theory's value with respect to the disease.

Generating such a gargantuan dataset for a practical-size cohort of at least a few thousand individuals is prohibitively expensive at this time (the genetics alone would require a budget of tens of millions of US dollars). Because these imaginary, ideal datasets are not available—and unlikely to be generated in the near future—the real-life heritability human disease estimates rely on (1) access to simplified-structure, cheaper datasets and (2) mathematical models with strong, simplifying assumptions permitting inference from incomplete data. There are three major groups of mathematical approaches—twin, pedigree, and genetic association studies—which constitute the modern toolbox for dissecting disease etiology.

Twin studies

This type of data includes limited phenotypic descriptors (disease or no disease) and data regarding genetic relatedness between twins (monozygotic or dizygotic, abbreviated as DZ and MZ), with 100% and 50% genetic similarity of MZ and DZ twins, respectively. With the strong assumption that the environment is identical for both individuals in each twin pair, we can then use a simple model to attribute disease status in concordance to the twins' genetic similarity.[7–11]

Pedigree studies

These limited phenotypic data include disease or no-disease statuses for each family member. Genetic data are reduced to a pedigree structure, in which siblings share half of the genetic variants with each parent and with each other. No explicit environmental data are provided. Model assumptions can include the same environment for all individuals described in the dataset (earlier models), or distinct shared environments for siblings, spouses, families, and individuals (in the more recent and sophisticated models).[12]

Whole-genome sequences and genetic association studies

In this case, the genetic data include individual-level genotypes for a large number of participants, possibly with knowledge of family structure for a subset of individuals. Phenotypic data involves disease status. Typically there are no environmental data available, and individual-specific environments are implicitly assumed to be identical for all participants.[13]

One should expect that each simplifying assumption in a mathematical heritability model results in biased parameter estimates. In twin analyses, all concordant disease states between twins are explained genetically. Therefore, we would expect that heritability estimates from twin data would be the highest. On the one hand, association studies focus on the explanatory power of common genetic variation—therefore, by design, heritabilities estimated with association data are smaller than estimates that would be obtained from total genetic variation (i.e., common plus rare plus ultra-rare/individual). On the other hand, association analysis typically ignores environmental data, which means that heritability estimates should be inflated with respect to hypothetical association studies with explicit environmental data included. Family pedigree analyses incorporate both limited genetic and limited environmental data and thus promise better-balanced heritability estimates. Keeping the strengths and weaknesses of the currently available tools in mind, we expect that richer data and more complex models are needed in order to dissect the influence of environment and genetics on human traits.

What we propose here

To represent genetics we will use family trees, as in the pedigree studies mentioned above. We will implicitly represent environmental influences in random effects, associated by the predetermined environmental relationship (familial, environment shared by couples, siblings, and families). More interestingly, we will construct models that consider gene-environment interactions and compare them with their counterparts in the absence of the mixed-effect regression's interaction term (Table 1). On the phenotypic side, we will use disease presence or absence status for a collection of diseases rather than a single disease. Furthermore, we will incorporate explicit environmental data—each family's geographic position will be associated with multidimensional environmental measurements, systematically collected over United States (US) territories. For each family we will also include the sociodemographic, economic, and topographic parameters linked to their geographic position. In addition, by incorporating families' geographic proximity in the model, we will be inferring implicit environmental variation that is not explained by explicit environmental measurements.

RESULTS

We fit the models in a Markov chain Monte Carlo procedure with a Bayesian framework (see STAR Methods) for the ten most common neuropsychiatric disorders in our data: anxiety phobic disorder, depression, migraine, adjustment disorder, substance abuse, attention-deficit hyperactivity disorder (ADHD), bipolar disorder, unspecified recurrent headaches, sleep disorder, and post-traumatic stress disorder (PTSD). Stratified by models, Table 2, shows the mean estimates of the heritability and environmental statistics, indicating how much of the outcome variation can be explained by the individual environment (e^2), the familial environment (f^2), the environment shared by couples (c^2) and siblings (s^2), and the shared geographic location of residence (p^2). Table 2 also provides how much variation can be accounted for by the interactions between the aforementioned environmental and genetic effects. The widely applicable information criterion (WAIC[14]) and Pareto-smoothed importance sampling leave-one-out cross-validation (PSIS-LOO-CV[15]) in Table 2 are Bayesian information criteria that estimate the out-of-sample prediction error. Like the more commonly known Akaike information criterion, AIC,[16] both the Bayesian WAIC and PSIS-LOO-CV reward goodness of fit, but penalize model complexity and large parameter space size. They are commonly used to compare a collection of competing models fit to the same data. Models with the smallest WAIC or PSIS-LOO-CV provide the optimum balance between complexity and explanatory powers.

CellPress
OPEN ACCESS

Table 1. Model setups and statistics

Model	Fixed effects	Random effects	Statistics
Linear model 0	Demo + Env	G + E	h^2, e^2
Linear model 1	Demo + Env	Geo + G + E	p^2, h^2, e^2
Interaction model 1	Demo + Env	Geo + G + E + GE	p^2, h^2, e^2, he^2
Linear model 2	Demo + Env	Geo + G + F + C + S + E	p^2, h^2, f^2, c^2, s^2, e^2
Interaction model 2	Demo + Env	Geo + G + F + C + S + E + GF + GC + GS + GE	p^2, h^2, f^2, c^2, s^2, e^2, hf^2, hc^2, hs^2, he^2

The fixed-effect terms are sex + age (Demo) and environmental quality indices (Env).
The random-effect terms are defined by the partition of the phenotype explained by the following. Geo, geographic position, described by coordinates (latitude and longitude); G, genetics; E, the individually independent environment; F, the environment shared by family members; C, the environment shared by couples; S, the environment shared by siblings; GE, the interaction between genetics and the individually independent environment; GF, the interaction between genetics and the family-shared environment; GC, the interaction between genetics and the couples-shared environment; GS, the interaction between genetics and the siblings-shared environment.

Figure 1 shows heritability estimates juxtaposed with model-specific WAIC values, which are also provided in Table 2. For each of the neuropsychiatric diseases we discussed, we plotted a model selection graph on the top panel, indicating the WAIC change along two forward selection traces (the green and golden points). The bar plot on the bottom panel of Figure 1 shows how much variance could be explained by each effect variable, grouped into four categories: heritability (gray bars), geographic location (violet bars), environmental factors (yellow-orange bars), and gene-environmental interactions (blue bars). We can draw the following conclusions from this analysis.

First, interaction models (IM1 and IM2) fit the data better than their linear counterparts (LM1 and LM2) in 17 out of 20 comparisons. The three exceptions are LM2 versus IM2 in adjustment disorder and substance abuse, and LM1 versus IM1 in bipolar disorder. The lower WAIC estimates suggest the interaction models fit the data better than the linear model, with smaller information loss.[14]

Correspondingly, gene-environment interactions seem to explain a significant portion of the total phenotypic variance. For example, gene-environment interactions under the best-fitting IM1 model explain over 29% of the total anxiety/phobic disorder phenotypic variance (blue bars in the upper left bar plot of Figure 1). Similarly, under the IM2 model, the interaction terms explain close to 30% of the total variance for unspecified recurrent headaches, sleep disorders, and PTSD.

By contrast, for some diseases, such as substance abuse and adjustment disorder, the gene-environment interactions do not appear to explain a significant portion of trait variance, so that interaction models are outcompeted by linear models according to WAIC and the associated confidence intervals.

Additionally, heritability estimates differ significantly between linear and interaction models. For ADHD, the simplest G + E (LM0) model corresponds to an extremely high heritability estimate (95%). However, the best (in terms of the WAIC model), IM2, incorporating all environmental and interaction effects, corresponds to a much lower heritability of 55%. Similarly, for bipolar disorder, heritability is as high as 80% for LM0, while for the WAIC-best model, IM2, it drops to 53%. This pattern is common across all of the neuropsychiatric disorders that we studied: The simpler models (LM0, LM1, and IM1) tend to provide higher heritability estimates compared with the more complex models (LM2 and IM2).

If we apply a different model selection criterion, called PSIS-LOO-CV,[17] model selection choices are exactly reproduced for all diseases except for the comparison between IM1 and IM2 in unspecified recurrent headache (Table 2). In the case of unspecified recurrent headache, the confidence intervals of PSIS-LOO-CV overlap for IM1 and IM2 models, suggesting that PSIS-LOO-CV cannot choose between these two interaction models. While WAIC estimates differ significantly between IM1 and IM2, the two model selection criteria give consistent choices, with PSIS-LOO-CV providing more uncertainty. We therefore believe that our results are robust.

Besides the findings regarding the G-by-E effects and heritabilities, estimates of p^2, the proportion of variance contributed by each family's geographic position, are remarkably variable across disorders. For anxiety/phobic disorder, migraine, substance abuse, and bipolar disorder, the geographic position does not explain much of the variance (under 2% in all models, including the WAIC-best model). By contrast, for ADHD and PTSD, the geographic variation contributes more profoundly to the overall variance. The p^2 estimates for ADHD and PTSD are close to 6%, according to their WAIC-best model LM2. Figures 2 and S1 show the geographic random effects' posterior mean estimates $f_p(\mathbf{x})$ (see Equation 8 in the models section of STAR Methods). Given the geographic coordinates vector $\mathbf{x}$, function $f_p(\mathbf{x})$ was described by a Gaussian process assuming adjacent geographic locations have close values.

For both ADHD and PTSD, all models across the complexity spectrum (LM1, IM1, LM2, and IM2) give nearly identical patterns of $f_p(\mathbf{x})$ estimates across the continental US (see Figure S1). This suggests that individuals living in the southern US and near the Great Lakes region may have been exposed to a higher risk of ADHD due to (as yet unidentified) geographic/environmental effects. Similarly, residents of the US west coast and the New England region bear higher PTSD. Note that we explicitly tried to account for geographically associated environmental factors by incorporating environmental quality indices (therefore, known air, water, and land pollutants have already been adjusted for [see Table 1 and the models section of STAR Methods]).

We also inspected the fixed-effect estimates of demographic (sex and age) and environmental factors (fixed environmental

Table 2. Mean estimates and 95% highest posterior density credible intervals of the heritability and environmental statistics (see Table S1 for details on simulations and inferences under the proposed models)

	p^2	h^2	f^2	c^2	s^2	e^2	hf^2	hc^2	hs^2	he^2	WAIC	PSIS-LOO-CV
Anxiety phobic disorder												
LM0	–	53.52 (44.94, 64.42)%	–	–	–	46.48 (35.58, 55.06)%	–	–	–	–	257,729.31 (256,459.62, 258,999.00)	291,289.56 (289,805.55, 292,773.57)
LM1	1.40 (0.96, 1.97)%	47.50 (43.82, 52.54)%	–	–	–	51.10 (45.92, 54.86)%	–	–	–	–	240,648.05 (239,482.50, 241,813.60)	284,715.61 (283,263.98, 286,167.24)
IM1	2.35 (1.53, 3.04)%	31.92 (20.40, 55.46)%	–	–	–	36.36 (27.03, 44.87)%	–	–	–	29.37 (14.29, 42.94)%	195,306.87 (194,145.39, 196,468.35)	263,091.00 (261,644.38, 264,537.62)
LM2	1.54 (0.98, 2.10)%	56.44 (50.26, 60.70)%	1.99 (0.32, 4.06)%	31.45 (27.93, 33.82)%	5.12 (1.70, 9.43)%	3.47 (0.50, 7.39)%	–	–	–	–	269,150.17 (267,800.73, 270,499.61)	296,226.08 (294,703.79, 297,748.37)
IM2	2.02 (1.29, 2.84)%	47.42 (26.90, 60.63)%	1.97 (0.08, 5.22)%	19.47 (12.41, 25.20)%	4.66 (0.61, 9.82)%	3.40 (0.29, 6.94)%	0.95 (0.00, 4.69)%	12.70 (4.31, 23.10)%	4.05 (0.00, 11.12)%	3.37 (0.00, 10.86)%	245,390.66 (244,122.11, 246,659.21)	284,535.98 (283,018.76, 286,053.20)
Depression												
LM0	–	69.48 (61.50, 79.38)%	–	–	–	30.52 (20.62, 38.50)%	–	–	–	–	176,007.77 (174,690.14, 177,325.40)	197,970.08 (196,439.77, 199,500.39)
LM1	2.55 (1.52, 3.65)%	68.76 (57.32, 82.22)%	–	–	–	28.69 (14.56, 40.43)%	–	–	–	–	178,182.13 (176,836.49, 179,527.77)	197,324.49 (195,796.14, 198,852.84)
IM1	4.69 (3.21, 6.02)%	55.35 (44.43, 69.13)%	–	–	–	12.87 (8.29, 19.87)%	–	–	–	27.10 (17.38, 38.25)%	139,055.19 (137,852.02, 140,258.36)	184,686.67 (183,193.74, 186,179.60)
LM2	2.07 (1.34, 2.94)%	49.53 (43.08, 55.48)%	8.57 (3.43, 12.94)%	35.00 (31.65, 37.78)%	2.97 (0.07, 7.40)%	1.86 (0.14, 5.47)%	–	–	–	–	143,671.22 (142,640.64, 144,711.80)	177,874.25 (176,496.13, 179,252.37)
IM2	2.30 (1.25, 3.58)%	50.30 (43.53, 57.38)%	7.12 (1.21, 15.90)%	31.18 (20.32, 38.22)%	1.68 (0.07, 4.59)%	1.99 (0.02, 5.57)%	0.49 (0.00, 1.76)%	1.98 (0.00, 11.24)%	0.77 (0.00, 2.57)%	2.19 (0.00, 7.99)%	135,787.57 (134,804.02, 136,771.12)	173,205.69 (171,849.96, 174,561.42)
Migraine												
LM0	–	57.31 (44.37, 74.73)%	–	–	–	42.69 (25.27, 55.63)%	–	–	–	–	179,692.88 (178,303.44, 181,082.32)	194,241.46 (192,718.76, 195,764.16)
LM1	0.60 (0.35, 0.89)%	48.47 (38.09, 60.94)%	–	–	–	50.93 (38.44, 61.42)%	–	–	–	–	165,004.39 (163,752.75, 166,256.03)	188,446.05 (186,970.39, 189,921.71)

(*Continued on next page*)

Table 2. *Continued*

	p^2	h^2	f^2	c^2	s^2	e^2	hf^2	hc^2	hs^2	he^2	WAIC	PSIS-LOO-CV
IM1	1.06 (0.63, 1.51)%	31.14 (17.40, 49.83)%	–	–	–	45.89 (33.71, 56.61)%	–	–	–	21.91 (8.35, 43.25)%	133,837.58 (132,839.27, 134,835.89)	177,214.09 (175,772.12, 178,656.06)
LM2	0.65 (0.35, 0.99)%	49.41 (38.69, 64.85)%	3.01 (0.32, 8.21)%	14.01 (9.56, 18.79)%	13.84 (4.89, 23.04)%	19.07 (4.49, 35.67)%	–	–	–	–	174,572.51 (173,229.81, 175,915.21)	192,363.95 (190,855.34, 193,872.56)
IM2	0.77 (0.42, 1.17)%	46.82 (35.62, 57.99)%	4.57 (0.48, 11.64)%	11.86 (4.53, 20.00)%	15.54 (5.05, 24.94)%	13.17 (0.68, 33.14)%	1.34 (0.00, 4.34)%	2.23 (0.00, 8.44)%	1.88 (0.00, 4.93)%	1.82 (0.00, 6.34)%	170,124.00 (168,807.66, 171,440.34)	191,132.71 (189,617.06, 192,648.36)
Adjustment disorder												
LM0	–	68.94 (60.06, 76.83)%	–	–	–	31.06 (23.17, 39.94)%	–	–	–	–	128,812.67 (127,578.50, 130,046.84)	146,767.54 (145,311.69, 148,223.39)
LM1	4.00 (2.61, 5.52)%	62.14 (53.13, 69.43)%	–	–	–	33.86 (26.15, 42.96)%	–	–	–	–	125,231.76 (124,031.40, 126,432.12)	144,010.34 (142,578.76, 145,441.92)
IM1	7.55 (5.08, 10.04)%	60.65 (41.43, 76.10)%	–	–	–	14.57 (2.27, 24.19)%	–	–	–	17.24 (2.02, 31.67)%	118,529.41 (117,371.56, 119,687.26)	142,509.46 (141,050.65, 143,968.27)
LM2	3.51 (2.47, 4.79)%	27.82 (17.26, 36.92)%	21.65 (15.43, 27.97)%	36.25 (31.03, 41.89)%	5.04 (0.28, 8.96)%	5.73 (0.04, 12.01)%	–	–	–	–	108,221.89 (107,194.65, 109,249.13)	131,849.40 (130,522.81, 133,175.99)
IM2	3.37 (1.90, 4.88)%	32.53 (22.77, 40.77)%	13.60 (5.21, 20.90)%	42.59 (32.21, 51.84)%	2.02 (0.16, 4.50)%	2.71 (0.03, 5.98)%	0.95 (0.00, 3.52)%	0.64 (0.00, 1.73)%	0.99 (0.00, 4.68)%	0.59 (0.00, 1.82)%	113,680.71 (112,586.79, 114,774.63)	134,470.01 (133,113.51, 135,826.51)
Substance abuse												
LM0	–	51.67 (40.51, 66.57)%	–	–	–	48.33 (33.43, 59.49)%	–	–	–	–	119,551.43 (118,226.49, 120,876.37)	128,775.41 (127,324.72, 130,226.10)
LM1	2.10 (0.98, 3.70)%	53.77 (37.89, 89.74)%	–	–	–	44.12 (6.73, 60.36)%	–	–	–	–	121,334.21 (119,979.12, 122,689.30)	128,047.10 (126,603.56, 129,490.64)
IM1	2.29 (1.07, 3.81)%	40.66 (31.59, 48.66)%	–	–	–	53.70 (37.49, 61.66)%	–	–	–	3.35 (0.00, 13.24)%	110,533.35 (109,318.80, 111,747.90)	126,149.23 (124,716.08, 127,582.38)
LM2	1.62 (0.96, 2.38)%	40.13 (32.24, 46.53)%	3.11 (0.20, 5.81)%	34.45 (29.05, 40.41)%	8.03 (2.64, 13.63)%	12.66 (1.95, 21.80)%	–	–	–	–	107,571.67 (106,400.26, 108,743.08)	123,469.25 (122,078.41, 124,860.09)

(*Continued on next page*)

Table 2. *Continued*

	p^2	h^2	f^2	c^2	s^2	e^2	hf^2	hc^2	hs^2	he^2	WAIC	PSIS-LOO-CV
IM2	2.31 (1.18, 3.66)%	41.65 (30.25, 53.14)%	3.62 (0.17, 8.70)%	26.16 (15.56, 36.32)%	8.49 (2.01, 15.28)%	6.79 (0.35, 14.17)%	1.58 (0.00, 5.55)%	7.58 (0.00, 17.21)%	1.06 (0.00, 3.60)%	0.75 (0.00, 2.88)%	111,049.17 (109,814.90, 112,283.44)	125,555.95 (124,121.80, 126,990.10)
ADHD												
LM0	–	95.22 (88.59, 99.88)%	–	–	–	4.78 (0.12, 11.41)%	–	–	–	–	90,855.79 (89,773.01, 91,938.57)	106,646.84 (105,399.46, 107,894.22)
LM1	3.48 (2.28, 4.60)%	88.19 (80.62, 94.62)%	–	–	–	8.33 (1.39, 15.59)%	–	–	–	–	89,893.81 (88,885.66, 90,901.96)	107,365.15 (106,110.44, 108,619.86)
IM1	3.52 (2.25, 4.91)%	76.58 (69.83, 83.63)%	–	–	–	4.36 (2.04, 10.07)%	–	–	–	15.53 (10.16, 21.20)%	75,706.35 (74,759.16, 76,653.54)	94,878.98 (93,761.72, 95,996.24)
LM2	3.49 (2.26, 4.94)%	48.23 (36.21, 58.38)%	24.60 (15.25, 39.05)%	17.15 (11.38, 21.71)%	1.67 (0.02, 3.48)%	4.85 (0.07, 13.24)%	–	–	–	–	89,568.83 (88,558.49, 90,579.17)	106,050.93 (104,810.11, 107,291.75)
IM2	5.80 (4.09, 8.00)%	55.22 (42.20, 67.84)%	3.22 (0.01, 9.37)%	2.45 (0.00, 10.93)%	1.72 (0.05, 4.94)%	1.48 (0.02, 3.44)%	8.66 (1.04, 20.44)%	21.11 (9.64, 30.64)%	0.14 (0.00, 0.68)%	0.21 (0.00, 1.14)%	57,894.43 (57,295.36, 58,493.50)	89,048.44 (88,006.33, 90,090.55)
Bipolar disorder												
LM0	–	79.66 (72.10, 87.38)%	–	–	–	20.34 (12.62, 27.90)%	–	–	–	–	65,719.01 (64,697.44, 66,740.58)	75,862.89 (74,636.60, 77,089.18)
LM1	0.97 (0.50, 1.52)%	77.43 (72.13, 86.46)%	–	–	–	21.61 (12.68, 27.25)%	–	–	–	–	64,106.44 (63,110.64, 65,102.24)	75,406.16 (74,191.33, 76,620.99)
IM1	1.50 (0.68, 2.44)%	81.57 (73.34, 90.50)%	–	–	–	4.76 (0.15, 12.39)%	–	–	–	12.18 (0.80, 22.00)%	65,983.85 (64,891.17, 67,076.53)	77,988.95 (76,723.30, 79,254.60)
LM2	0.94 (0.49, 1.52)%	45.68 (27.56, 61.37)%	16.49 (6.45, 25.97)%	21.96 (11.29, 28.34)%	3.65 (0.28, 7.20)%	11.27 (0.48, 26.99)%	–	–	–	–	58,846.71 (57,951.28, 59,742.14)	72,074.67 (70,913.27, 73,236.07)
IM2	2.18 (1.02, 3.67)%	53.22 (25.43, 73.22)%	4.27 (0.14, 10.92)%	6.60 (0.01, 20.06)%	2.02 (0.02, 5.92)%	3.16 (0.02, 9.74)%	4.04 (0.00, 12.78)%	22.43 (7.43, 34.23)%	0.42 (0.00, 1.74))%	1.64 (0.00, 6.69)%	36,754.75 (36,228.37, 37,281.13)	60,323.29 (59,349.95, 61,296.63)
Unspecified recurrent headaches												
LM0	–	52.90 (24.75, 96.63)%	–	–	–	47.10 (3.37, 75.25)%	–	–	–	–	63,569.71 (62,403.35, 64,736.07)	65,366.62 (64,182.07, 66,551.17)

(*Continued on next page*)

Table 2. *Continued*

	p^2	h^2	f^2	c^2	s^2	e^2	hf^2	hc^2	hs^2	he^2	WAIC	PSIS-LOO-CV
LM1	2.34 (1.51, 3.39)%	40.78 (31.76, 51.20)%	–	–	–	56.88 (46.03, 66.40)%	–	–	–	–	55,287.67 (54,290.48, 56,284.86)	64,434.32 (63,281.10, 65,587.54)
IM1	6.23 (4.26, 8.83)%	23.77 (6.32, 55.84)%	–	–	–	33.96 (0.70, 55.89) %	–	–	–	36.04 (24.66, 43.17)%	40,592.46 (39,731.29, 41,453.63)	52,606.16 (51,683.98, 53,528.34)
LM2	2.25 (1.43, 3.23)%	26.62 (8.83, 42.34)%	8.10 (0.34, 15.60)%	18.24 (6.89, 28.40)%	18.79 (2.90, 32.95)%	26.00 (6.99, 43.40)%	–	–	–	–	55,280.79 (54,321.10, 56,240.48)	63,910.85 (62,759.02, 65,062.68)
IM2	5.48 (3.30, 7.65)%	17.68 (3.69, 39.63)%	3.52 (0.04, 8.36)%	11.98 (0.01, 25.74)%	10.57 (0.03, 22.92, 14.11)%	14.11 (0.74, 27.30)%	0.92 (0.00, 4.20)%	19.91 (5.66, 38.30)%	6.83 (0.00, 23.99)%	8.99 (0.00, 25.35)%	32,654.79 (32,126.08, 33,183.50)	53,238.40 (52,274.86, 54,201.94)
Sleep disorder												
LM0	–	47.50 (33.86, 61.38)%	–	–	–	52.50 (38.62, 66.14)%	–	–	–	–	60,866.74 (59,747.27, 61,986.21)	65,015.00 (63,799.64, 66,230.36)
LM1	2.11 (1.29, 2.99)%	40.15 (30.78, 50.45)%	–	–	–	57.74 (46.95, 67.26)%	–	–	–	–	55,129.58 (54,075.63, 56,183.53)	61,872.96 (60,726.07, 63,019.85)
IM1	5.55 (3.45, 7.70)%	13.61 (1.71, 28.47)%	–	–	–	45.90 (32.31, 60.71)%	–	–	–	34.93 (18.56, 50.17)%	37,786.65 (37,132.72, 38,440.58)	56,917.80 (55,828.63, 58,006.97)
LM2	1.72 (1.07, 2.41)%	30.80 (19.42, 40.54)%	4.26 (0.51, 9.21)%	23.13 (16.25, 30.14)%	12.99 (2.71, 24.09)%	27.09 (17.05, 40.25)%	–	–	–	–	49,665.71 (48,785.67, 50,545.75)	59,973.66 (58,857.58, 61,089.74)
IM2	4.16 (2.22, 6.17)%	24.29 (2.33, 48.33)%	3.73 (0.03, 9.92)%	7.83 (0.01, 22.19)%	13.66 (3.14, 26.56)%	14.11 (3.33, 36.75)%	0.38 (0.00, 1.75)%	24.22 (6.00, 40.01)%	3.82 (0.00, 10.80)%	3.80 (0.00, 14.40)%	34,098.02 (33,515.27, 34,680.77)	53,307.66 (52,302.53, 54,312.79)
PTSD												
LM0	–	65.28 (52.03, 85.61)%	–	–	–	34.72 (14.39, 47.97)%	–	–	–	–	30,127.48 (29,251.32, 31,003.64)	32,319.99 (31,361.90, 33,278.08)
LM1	3.10 (1.17, 5.79)%	63.15 (47.25, 79.69)%	–	–	–	33.75 (16.16, 50.53))%	–	–	–	–	29,902.80 (29,032.46, 30,773.14)	32,125.35 (31,172.61, 33,078.09)
IM1	7.71 (3.36, 13.88)%	42.44 (18.04, 64.50)%	–	–	–	11.38 (0.07, 30.44)%	–	–	–	38.48 (28.64, 49.33)%	15,312.56 (14,903.66, 15,721.46)	25,411.63 (24,645.78, 26,177.48)

(*Continued on next page*)

Table 2. *Continued*

	p^2	h^2	f^2	c^2	s^2	e^2	hf^2	hc^2	hs^2	he^2	WAIC	PSIS-LOO-CV
LM2	2.51 (0.95, 4.66)%	28.30 (10.50, 44.30)%	16.92 (7.78, 25.64)%	25.84 (17.80, 33.92)%	13.21 (3.44, 23.84)%	13.22 (2.63, 25.75)%	–	–	–	–	25,494.21 (24,771.62, 26,216.80)	30,175.70 (29,285.47, 31,065.93)
IM2	6.44 (2.84, 10.82)%	30.43 (2.81, 53.74)%	5.14 (0.04, 15.64)%	6.09 (0.01, 20.57)%	5.61 (0.17, 12.64)%	3.93 (0.03, 9.87)%	1.50 (0.00, 6.09)%	36.29 (23.24, 54.61)%	2.90 (0.00, 12.62)%	1.67 (0.00, 8.40)%	10,777.49 (10,510.50, 11,044.48)	18,752.11 (18,208.68, 19,295.54)

ADHD, attention-deficit hyperactivity disorder; PTSD, post-traumatic stress disorder.

The statistics are defined by the partition of the phenotype explained by the following. p^2, geographic position, described by coordinates (latitude and longitude); h^2, genetics; e^2, the individually independent environment; f^2, the environment shared by family members; c^2, the environment shared by couples; s^2, the environment shared by siblings; he^2, the interaction between genetics and the individually independent environment; hf^2, the interaction between genetics and the family-shared environment; hc^2, the interaction between genetics and the couples-shared environment; hs^2, the interaction between genetics and the siblings-shared environment. WAIC and PSIS-LOO-CV data are presented with 95% confidence intervals.

The widely applicable information criterion (WAIC) and Pareto-smoothed importance sampling leave-one-out cross-validation (PSIS-LOO-CV) reward goodness of fit but penalize more complex models. The lower the WAIC or PSIS-LOO-CV, the better the model.

quality index [EQI] scores) in our data for these ten most common neuropsychiatric diseases. Figure 3 shows the posterior distribution of the log-odds (logit) change contributed by one's sex according to each disease's WAIC-best model. We code females in zero and males in one, so a greater log-odds change suggests a higher risk in males. After controlling for other effects, we observed high risks of ADHD and substance abuse in males and high risks of migraine, unspecified recurrent headaches, and PTSD in females (Figure 3). Similarly, Figure 4 summarizes the log-odds change contributed by the numeric age according to each disease's WAIC-best model. Higher estimates of fixed effects in Figure 4 indicate higher risks associated with older age; for example, sleep disorders are more prevalent in the older population. Negative values of the estimate, as obtained for ADHD and bipolar disorder, indicate that these disorders are more common in the younger population (Figure 4).

We then analyzed how the fixed-effect environmental qualities affect our neuropsychiatric diseases' log-odds. Figure S2 shows the nonlinear effects estimated for the five EQI domains (air, water, land, sociodemographic, and built environment) based on each disease's WAIC-best model. We summarized and standardized each EQI domain by a principal component analysis (PCA) procedure into one-dimensional PCA scores. Higher scores generally reflect worse environmental quality. To visualize the result, we converted the PCA scores to percentiles based on the score distribution in the whole population and used the 2.5th to 97.5th percentiles as the x axis limits of the plots. The blue segments of the lines indicate EQI regions that do not change the log-odds significantly. The olive segments are EQI regions that influence the disease log-odds significantly. The figures demonstrate the miscellaneous effects of various environmental qualities. For air quality, worse environments (high scores) may be associated with increased risks of depression, adjustment disorder, ADHD, and bipolar disorder (first column of Figure S2). On the contrary, residents in areas of worse air quality seem to have lower risks of anxiety phobic disorder, unspecified recurrent headaches, sleep disorder, and PTSD (first column of Figure S2). Compared with air quality, water quality has only minor effects on the studied neuropsychiatric diseases' log-odds (second column in Figure S2). Additionally, lower sociodemographic quality scores may be associated with elevated risks of depression, adjustment disorder, and ADHD, as well as decreased risk of unspecified recurrent headaches (fourth column in Figure S2). The last column of Figure S2 suggests the broad impact of built-environmental quality, which appears linked to significantly higher risks of depression, adjustment disorder, substance abuse, ADHD, bipolar disorder, and sleep disorder.

DISCUSSION

For selectionists working with domesticated species the notion of heritability is an instrument, while the remaining environmental variability is a nuisance. In the case of human disease, the situation may be quite the opposite. Associations between genetic variation and disease may lead to genetic tests and indicate molecular pathways that should be targeted for treatment. However, for most complex diseases, genetic variation is not very instrumental in disease treatment; predicted genetic

CellPress
OPEN ACCESS

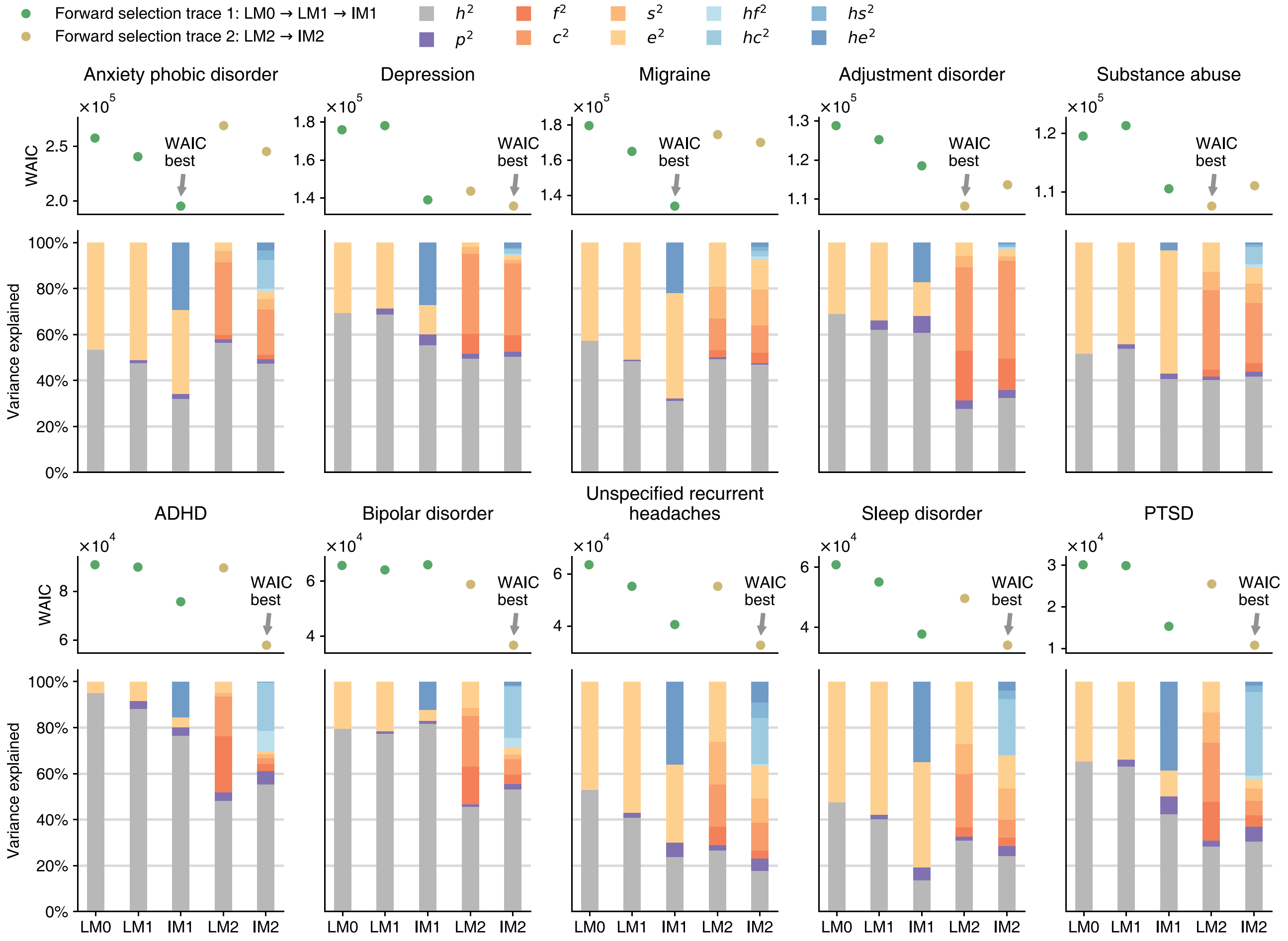

Figure 1. Model WAIC estimates and the mean estimates of heritability and environmental statistics

Bar plots show the posterior mean heritability estimates (h^2, gray), variance explained by the geographic location (p^2, violet), variances explained by shared environments (f^2, familial; c^2, couple-shared; s^2, sibling-shared: yellow/orange-colored bars), and variances explained by gene-environment interactions (hf^2, gene-familial; hc^2, gene-couple-shared-environmental; hs^2, gene-sibling-shared environmental: blue-colored bars) given in the five models for the ten most diagnosed neuropsychiatric diseases in our data. LM0, LM1, and LM2 are the three linear models that consider only the additive effects of genetics and shared environments as defined in Table 1. IM1 and IM2 are the two interaction models. Corresponding to their linear counterpart (LM1 and LM2), these two models consider the gene-environmental interactions as defined in Table 1. The five models form two forward selection traces: Linear model 0 (LM0) $\vec{x}$ Linear model 1 (LM1) $\vec{x}$ Interaction model 1 (IM1) and Linear model 2 (LM2) $\vec{x}$ Interaction model 2 (IM2). Within each trace, the later models encompass all the preceding models' variables. The widely applicable information criterion (WAIC) rewards goodness of fit but penalizes more complex models. The lower the WAIC, the better the model. The scatterplot above each bar plot illustrates which model could be considered as the best one for each disease.

predisposition to complex disease is often seen as a verdict rather than an actionable warning or advice.

This is not so with environmental predisposition to disease. If we knew a certain environmental stimulus might trigger a particular disease, we could take preventive measures or design life strategies aimed at avoiding dangerous triggers. Similarly, with interactions of genetic and environmental signals, information can become practical and clinically actionable. If we were able to ascertain a catalog of genetic variants interacting with specific environmental stimuli, we could design personalized environmental plans for patients at risk.

For the ten most frequently diagnosed neuropsychiatric conditions in our data, we designed a group of Bayesian regression models incorporating various genetic, environmental, and demographic effects. By comparing models with G-by-E interactions with their linear counterparts, we established the existence of interactions with considerable strength between the genetic variations and environmental stimuli affecting the manifestation of neuropsychiatric disorders. We also estimated a set of model parameters measuring the strength of association between the patient's geographic location, sex, age, and the local environment's quality with the likelihood of disease manifestation.

Unexplained geographic variations (see Figures 2 and S1) can serve as a basis for future association studies. For example, we can scan myriad environmental factors to explain the difference in ADHD rates in California versus Georgia. Furthermore, it is reassuring to "rediscover" known factors that affect the phenotypic variation in neuropsychiatric disorders. For example,

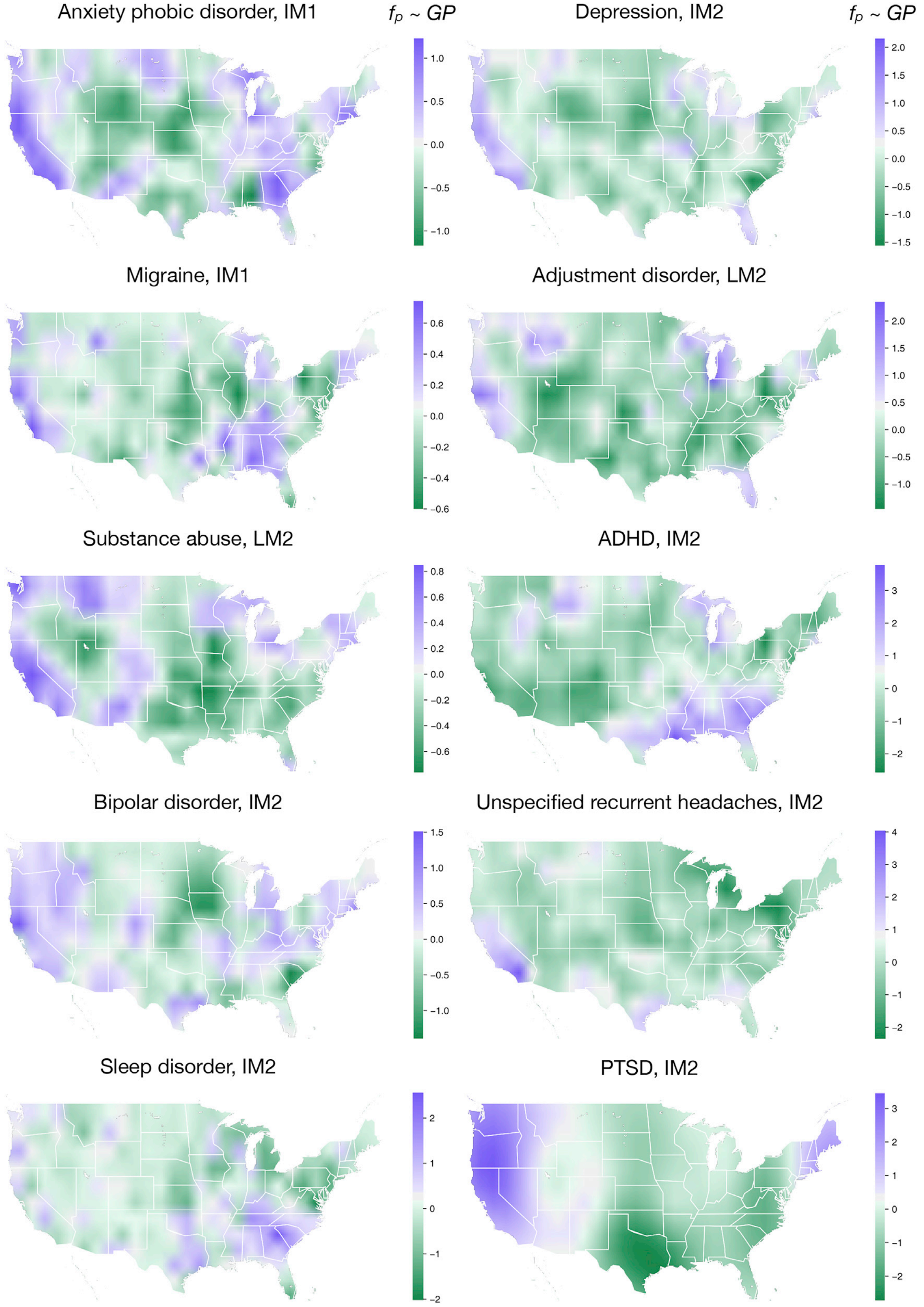

(legend on next page)

ADHD is associated with a younger age, while sleep disorder is a feature in much older people (see Figure 4).[18,19] Similarly, sex bias in diseases is well reported: migraine affects females more often while substance abuse is biased toward males (see Figure 3).[20–23]

Fixed effects estimated for EQIs (see Figure S2) indicate curious associations between the quality of the immediate environment and the rate of neuropsychiatric disorders. In the observational setting of our analyses, we can talk about environmental quality's predictive properties affecting disease rates but avoid any statements implying causality. For example, we can observe that deteriorating air quality is associated with increased rates of ADHD and decreased rates of sleep disorders. Specially designed observational studies (for example, involving an instrumental variable) might closely approximate randomized clinical trial causality analysis, but we will defer this to future follow-up studies.

Our analyses open additional research opportunities. One possible step forward would be to validate these findings in large population biobanks with genetic data (such as UK Biobank or FinnGen). The present work has demonstrated that much is waiting for investigation. We can compute the interactions between genetics and concrete environmental indices, such as geographic locations, pollutants, and socioeconomic factors. This analysis could produce more easily interpretable results than ours; we only considered interactions between random genetic and environmental effects that are more difficult to interpret. Moreover, we can exploit experiments and sequencing data to dig deeper into interactions between particular genetic variants and exterior conditions. By designing specific assays and methods, it is also possible to determine these interactions' directions and mechanisms. Does the G act as a modifier of the environmental effects, E? Does the E act as a modifier of the G? Are genetic epistasis (G-by-G) and E-by-E effects involved in disease etiology? Future efforts could answer these questions and allow us to map out strategic plans for preventive and precision medicine.

Limitations of the study

Our study finds substantial gene-environment interactions in common neuropsychiatric diseases through elegant methods. However, there remain many unsolved puzzles.

Data and methods

Our estimation method relies on the presumption that the pedigree and residency information truthfully reflect the real situation. In the current version of IBM's Health MarketScan database, demographic and family information is limited to age, sex, area of residence, and information about family members enrolled under the same health insurance policy. The data only label if a family member is a "parent" or a "child"—adoption or biological relation has not been meticulously documented. We also do not know how the residency information truthfully reflects the actual environmental exposure. This inconsistency between our assumptions and the information in data (or lack thereof) might compromise our estimation accuracy, even with the large scale of our data and the quality control steps we have performed. Our analysis and corresponding results are based on populations that consistently lived in the same geographic location during the study period. We excluded from our analysis a relatively small subset of families who lived in more than one place during the study period. Environmental exposures for such populations were more difficult to estimate and incorporate into our model. Therefore, caution should be exercised when interpreting and generalizing the results reported in this study.

On the methodology side, we relied on a fully Bayesian approach that provided merits such as a model-centered inference process, intuitive uncertainty quantification, and the availability of versatile model comparisons and regularization tools. However, a Bayesian method also entails many disadvantages. A Bayesian sampling only guarantees convergence to the posterior distribution, as its iteration time continues to infinity. For real-world complex models, various advanced tools such as multiple independent samplings, variational inference initialization, and No-U-Turn samplers[24] can be implemented to avoid instability and accelerate convergence (see the Bayesian inference section of STAR Methods for a more in-depth discussion about how we adapted the method to better approximate the variables). However, it is still difficult to tell how many steps we need to find an adequate sampling with an acceptable error. For complicated problems like ours, Bayesian methods also demand significant computational resources; for example, they require large memory for sampling storage. The process is also probably slower than frequentist maximum-likelihood approaches because Bayesian sampling requires repeated random number generation, which is not typically parallelizable and optimized in a regular computer.

Results interpretation

It is not definite what, precisely, the G-by-E effects represent. One well-liked interpretation reads the gene-environmental interaction as heterogeneous genetic effects conditioned on different environments or exterior exposures.[25–27] This interpretation provides a solution for the "missing heritability" puzzle, given that the G-by-E effect may comprise a considerable part of the heritable factor, undetectable in former linear models.[26,27] The one-directional interpretation (E influences G, heterogeneous genetics in response to the environment) is far from the only plausible interpretation. Conversely, it is also possible that genetics may affect the environment or the exterior exposure. Certain genotypes might influence one's preference for diets and living conditions. People are also continuously transforming

Figure 2. Mean estimates of the geographic random effects under best-fit model for each disease

These figure plots show the posterior mean estimates of the geographic random effects for each disease's WAIC-best model ($f_p(\mathbf{x})$) (see Equation 8 in the models section of STAR Methods). We modeled the geographic random effects $f_p(\mathbf{x})$ using a Gaussian process assuming that adjacent geographic locations have close-value random effects (assumption of smoothness). We did include residents of Hawaii and Alaska in our estimation process, but the results are not shown here. We omitted these results because the discontinuity between the geographic locations disobeys the Gaussian process's presumptions. The Gaussian process's poor extrapolation power also makes it difficult to estimate the random effects related to outlying states such as Alaska and Hawaii. Our data do not record any residents of other non-continental US islands.

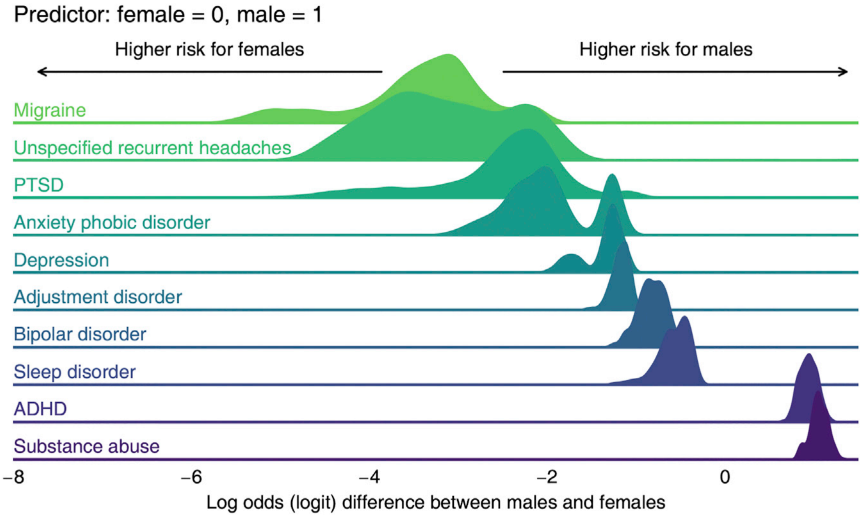

Figure 3. Posterior distribution of the log-odds (logit) change contributed by one's sex according to each disease's WAIC-best model

For each disease's WAIC-best model, this figure delineates the posterior distribution of the regression coefficient estimate associated with the dummy-codes sex (female = 0, male = 1). Because we used a logit link, the coefficient estimate represents the log-odds difference of the risk (in terms of diagnosis probability) between males and females. High values in this figure indicate high risks for males, and low values indicate high risks for females, after controlling for other effects in the regression.

their surrounding environments, in which process behavior-related genes might be involved. The most important clinical ramification of our analysis is that it should be possible to identify genetic variations that render people vulnerable to specific environmental stimuli. This is clinically relevant because it opens avenues to personalized preventive medical interventions.

The reader might wonder how to interpret high values of hc^2 for bipolar disorder, PTSD, and ADHD, coupled with reduced estimates of heritability. Essentially, the model and data "tell us" that, in nuclear families, parents (couple-specific, gene-environment interaction, denoted with subscript *c*) are more similar genetically and environmentally than randomly expected for specific disorders, such as PTSD and ADHD. For PTSD this may mean that both parents, but not offspring, might have been exposed to similar stressors, resulting in PTSD. Another reason for high hc^2 could be assortative mating: data suggest that couples tend to have similar psychological trends, such as ADHD. They are also possibly more genetically similar with each other than a randomly drawn couple. Because heritability is an additive genetics' portion of the overall phenotypic variance, increase in hc^2 necessarily reduces estimates in h^2 (heritability).

In this study, we estimated only the linear G-by-E effects with a most simplified model (one genetic variant by one environmental input), estimating their total input into the phenotypic variation, without the ability to identify specific genetic variants and environmental factors. It is plausible—and even likely—that higher orders of factor interactions may play a role in phenotypic variations. For example, genetic epistasis and environment-environmental effects could "collaborate" to produce G-by-G-by-E or G-by-E-by-E interaction effects. The next important target would be to map specific genetic variant-environmental stimulus pairs for each disorder and ascertain the mechanism(s) of these interactions. Achieving this goal is likely to require the construction of new datasets approaching the "ideal" dataset described in this article's introduction or bench experiments focusing on particular genes.

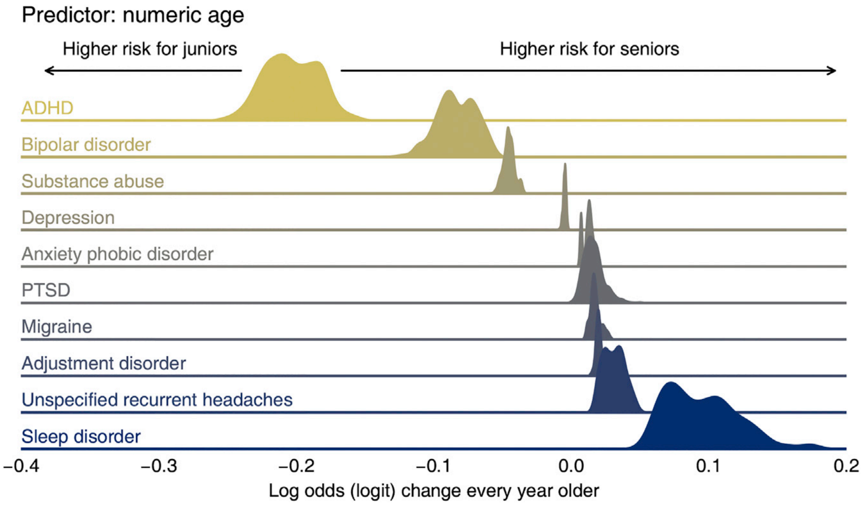

Figure 4. Posterior distribution of the log-odds (logit) change contributed by patient's numeric age according to each disease's WAIC-best model

For each disease's WAIC-best model, this figure delineates the posterior distribution of the regression coefficient estimate associated with numeric age. Because we used a logit link, the coefficient estimate represents the log-odds' change of the risk (in terms of the probability of diagnosis) at every year older. High values in this figure indicate high risks for seniors, and low values indicate high risks for juniors, after controlling for other effects in the regression.

As possibly the most famous saying among statisticians goes, "all models are wrong, but some are useful;"[28] our models are no exception. They possess explicit assumptions and implementation choices leading to potential biases, but they also significantly improve on the traditional, simpler models riddled with even stronger assumptions. In terms of assumptions, we introduced a series of increasing-fidelity models, mentioned in both the introduction and STAR Methods. Of course, even the most complicated set of assumptions that we use here is necessarily an abstracted version of a much more complicated real process. In terms of implementation, we made a series of choices with possibly detrimental consequences. For example, to estimate the posterior distribution over model parameter values, we implemented a Bayesian Markov chain Monte Carlo procedure. This implementation incorporated multiple checks for convergence of the Markov chain (reaching the limiting stationary distribution, where the frequencies of the parameter values closely approximate the true posterior probability). The procedure that we used is proven to be theoretically sound in principle, but because the convergence is guaranteed only by its theoretical limit (after an infinite number of iterations have passed), we could not guarantee that our model properly estimated posterior distributions for all parameters.

STAR★METHODS

Detailed methods are provided in the online version of this paper and include the following:

- KEY RESOURCES TABLE
- RESOURCE AVAILABILITY
  - Lead contact
  - Materials availability
  - Data and code availability
- EXPERIMENTAL MODEL AND SUBJECT DETAILS
- METHOD DETAILS
  - Data and study population
  - Modeling
- QUANTIFICATION AND STATISTICAL ANALYSIS
  - Models
  - Bayesian inference
  - Priors
  - Sampling
  - Statistical simulations of inference under the assumed model

SUPPLEMENTAL INFORMATION

Supplemental information can be found online at https://doi.org/10.1016/j.xcrm.2022.100736.

ACKNOWLEDGMENTS

The authors are grateful to M. Rzhetsky and E. Gannon for comments on early versions of the manuscript. This work was funded by the DARPA Big Mechanism program under ARO contract W911NF1410333 (A.R.), National Institutes of Health grants R01HL122712 (A.R.), 1P50MH094267 (A.R.), and U01HL108634-01 (A.R.), and a gift from Liz and Kent Dauten (A.R.).

AUTHOR CONTRIBUTIONS

H.Z. and A.R. formulated the study idea; H.Z., A.K., and A.R. performed the analysis; H.Z. and A.R. wrote the manuscript; H.Z., A.K., and A.R. edited the manuscript.

DECLARATION OF INTERESTS

The authors declare no competing interests.

Received: June 18, 2021
Revised: November 28, 2021
Accepted: August 12, 2022
Published: September 6, 2022

REFERENCES

1. Tropf, F.C., Lee, S.H., Verweij, R.M., Stulp, G., van der Most, P.J., de Vlaming, R., Bakshi, A., Briley, D.A., Rahal, C., Hellpap, R., et al. (2017). Hidden heritability due to heterogeneity across seven populations. Nat. Hum. Behav. *1*, 757–765. https://doi.org/10.1038/s41562-017-0195-1.
2. Sulc, J., Mounier, N., Günther, F., Winkler, T., Wood, A.R., Frayling, T.M., Heid, I.M., Robinson, M.R., and Kutalik, Z. (2020). Quantification of the overall contribution of gene-environment interaction for obesity-related traits. Nat. Commun. *11*, 1385. https://doi.org/10.1038/s41467-020-15107-0.
3. McAllister, K., Mechanic, L.E., Amos, C., Aschard, H., Blair, I.A., Chatterjee, N., Conti, D., Gauderman, W.J., Hsu, L., Hutter, C.M., et al. (2017). Current challenges and new opportunities for gene-environment interaction studies of complex diseases. Am. J. Epidemiol. *186*, 753–761. https://doi.org/10.1093/aje/kwx227.
4. Assary, E., Vincent, J.P., Keers, R., and Pluess, M. (2018). Gene-environment interaction and psychiatric disorders: review and future directions. Semin. Cell Dev. Biol. *77*, 133–143. https://doi.org/10.1016/j.semcdb.2017.10.016.
5. Fu, J., Nogueira, S.V., Drongelen, V.V., Coit, P., Ling, S., Rosloniec, E.F., Sawalha, A.H., and Holoshitz, J. (2018). Shared epitope-aryl hydrocarbon receptor crosstalk underlies the mechanism of gene-environment interaction in autoimmune arthritis. Proc. Natl. Acad. Sci. USA *115*, 4755–4760. https://doi.org/10.1073/pnas.1722124115.
6. Rivera, N.V., Patasova, K., Kullberg, S., Diaz-Gallo, L.M., Iseda, T., Bengtsson, C., Alfredsson, L., Eklund, A., Kockum, I., Grunewald, J., and Padyukov, L. (2019). A gene-environment interaction between smoking and gene polymorphisms provides a high risk of two subgroups of sarcoidosis. Sci. Rep. *9*, 18633. https://doi.org/10.1038/s41598-019-54612-1.
7. Arbet, J., McGue, M., and Basu, S. (2020). A robust and unified framework for estimating heritability in twin studies using generalized estimating equations. Stat. Med. *39*, 3897–3913. https://doi.org/10.1002/sim.8564.
8. Grasby, K.L., Verweij, K.J.H., Mosing, M.A., Zietsch, B.P., and Medland, S.E. (2017). Estimating heritability from twin studies. Methods Mol. Biol. *1666*, 171–194. https://doi.org/10.1007/978-1-4939-7274-6_9.
9. Scheike, T.H., Holst, K.K., and Hjelmborg, J.B. (2014). Estimating heritability for cause specific mortality based on twin studies. Lifetime Data Anal. *20*, 210–233. https://doi.org/10.1007/s10985-013-9244-x.
10. Verweij, K.J.H., Mosing, M.A., Zietsch, B.P., and Medland, S.E. (2012). Estimating heritability from twin studies. Methods Mol. Biol. *850*, 151–170. https://doi.org/10.1007/978-1-61779-555-8_9.
11. Lopes, M.C., Andrew, T., Carbonaro, F., Spector, T.D., and Hammond, C.J. (2009). Estimating heritability and shared environmental effects for refractive error in twin and family studies. Invest. Ophthalmol. Vis. Sci. *50*, 126–131. https://doi.org/10.1167/iovs.08-2385.

12. Bochud, M. (2017). Estimating heritability from nuclear family and pedigree data. Methods Mol. Biol. *1666*, 195–210. https://doi.org/10.1007/978-1-4939-7274-6_10.
13. Evans, L.M., Tahmasbi, R., Vrieze, S.I., Abecasis, G.R., Das, S., Gazal, S., Bjelland, D.W., de Candia, T.R., Haplotype Reference Consortium; and Goddard, M.E., et al. (2018). Comparison of methods that use whole genome data to estimate the heritability and genetic architecture of complex traits. Nat. Genet. *50*, 737–745. https://doi.org/10.1038/s41588-018-0108-x.
14. Watanabe, S. (2010). Asymptotic equivalence of Bayes cross validation and widely applicable information criterion in singular learning theory. Preprint at arXiv. https://doi.org/10.48550/arXiv.1004.2316.
15. Vehtari, A., Gelman, A., and Gabry, J. (2017). Practical Bayesian model evaluation using leave-one-out cross-validation and WAIC. Stat. Comput. *27*, 1413–1432.
16. Akaike, H. (1974). A new look at the statistical model identification. IEEE Trans. Automat. Contr. *19*, 716–723. https://doi.org/10.1109/TAC.1974.1100705.
17. Magnusson, M., Andersen, M.R., Jonasson, J., and Vehtari, A. (2020). Leave-one-out cross-validation for Bayesian model comparison in large data. Preprint at arXiv. https://doi.org/10.48550/arXiv.2001.00980.
18. Chung, W., Jiang, S.-F., Paksarian, D., Nikolaidis, A., Castellanos, F.X., Merikangas, K.R., and Milham, M.P. (2019). Trends in the prevalence and incidence of attention-deficit/hyperactivity disorder among adults and children of different racial and ethnic groups. JAMA Netw. Open *2*, e1914344.
19. Chong, Y., Fryar, C.D., and Gu, Q. (2013). Prescription Sleep Aid Use Among Adults: United States, 2005-2010 (US Department of Health and Human Services, Centers for Disease Control).
20. Maleki, N., Linnman, C., Brawn, J., Burstein, R., Becerra, L., and Borsook, D. (2012). Her versus his migraine: multiple sex differences in brain function and structure. Brain *135*, 2546–2559.
21. Vetvik, K.G., and MacGregor, E.A. (2017). Sex differences in the epidemiology, clinical features, and pathophysiology of migraine. Lancet Neurol. *16*, 76–87.
22. Brady, K.T., Grice, D.E., Dustan, L., and Randall, C. (1993). Gender differences in substance use disorders. Am. J. Psychiatry *150*, 1707–1711.
23. Merikangas, K.R., and McClair, V.L. (2012). Epidemiology of substance use disorders. Hum. Genet. *131*, 779–789.
24. Hoffman, M.D., and Gelman, A. (2014). The No-U-Turn sampler: adaptively setting path lengths in Hamiltonian Monte Carlo. J. Mach. Learn. Res. *15*, 1593–1623.
25. Purcell, S. (2002). Variance components models for gene–environment interaction in twin analysis. Twin Res. *5*, 554–571.
26. Manuck, S.B., and McCaffery, J.M. (2014). Gene-environment interaction. Annu. Rev. Psychol. *65*, 41–70.
27. Tropf, F.C., Lee, S.H., Verweij, R.M., Stulp, G., Van Der Most, P.J., De Vlaming, R., Bakshi, A., Briley, D.A., Rahal, C., Hellpap, R., et al. (2017). Hidden heritability due to heterogeneity across seven populations. Nat. Hum. Behav. *1*, 757–765.
28. Box, G.E.P. (1976). Science and statistics. J. Am. Stat. Assoc. *71*, 791–799. https://doi.org/10.1080/01621459.1976.10480949.
29. IBM Watson Health (2019). IBM MarketScan Research Databases.
30. Messer, L.C., Jagai, J.S., Rappazzo, K.M., and Lobdell, D.T. (2014). Construction of an environmental quality index for public health research. Environ. Health. *13*, 39. https://doi.org/10.1186/1476-069X-13-39.
31. Lobdell, D.T., Jagai, J.S., Rappazzo, K., and Messer, L.C. (2011). Data sources for an environmental quality index: availability, quality, and utility. Am. J. Public Health *101*, S277–S285. https://doi.org/10.2105/AJPH.2011.300184.
32. Morrell, C.H. (1998). Likelihood ratio testing of variance components in the linear mixed-effects model using restricted maximum likelihood. Biometrics, 1560–1568.
33. Duchateau, L., Janssen, P., and Rowlands, J. (1998). Linear Mixed Models. An Introduction with Applications in Veterinary Research (ILRI (Aka ILCA and ILRAD)).
34. Corbeil, R.R., and Searle, S.R. (1976). Restricted maximum likelihood (REML) estimation of variance components in the mixed model. Technometrics *18*, 31–38.
35. Boedeker, P. (2017). Hierarchical linear modeling with maximum likelihood, restricted maximum likelihood, and fully Bayesian estimation. Practical Assess. Res. Eval. *22*, 2.
36. Ly, A., Marsman, M., Verhagen, J., Grasman, R.P., and Wagenmakers, E.-J. (2017). A tutorial on Fisher information. J. Math. Psychol. *80*, 40–55.
37. Efron, B., and Hinkley, D.V. (1978). Assessing the accuracy of the maximum likelihood estimator: observed versus expected Fisher information. Biometrika *65*, 457–483.
38. Williams, P.M. (1995). Bayesian regularization and pruning using a Laplace prior. Neural Comput. *7*, 117–143.
39. Van Erp, S., Oberski, D.L., and Mulder, J. (2019). Shrinkage priors for Bayesian penalized regression. J. Math. Psychol. *89*, 31–50.
40. Carvalho, C.M., Polson, N.G., and Scott, J.G. (2009). Handling Sparsity via the Horseshoe, pp. 73–80.
41. Chung, Y., Rabe-Hesketh, S., Dorie, V., Gelman, A., and Liu, J. (2013). A nondegenerate penalized likelihood estimator for variance parameters in multilevel models. Psychometrika *78*, 685–709. https://doi.org/10.1007/s11336-013-9328-2.
42. Gelman, A. (2019). Prior Choice Recommendations.
43. Kucukelbir, A., Tran, D., Ranganath, R., Gelman, A., and Blei, D.M. (2017). Automatic differentiation variational inference. J. Mach. Learn. Res. *18*, 430–474.
44. Starke, L., and Ostwald, D. (2017). Variational Bayesian parameter estimation techniques for the general linear model. Front. Neurosci. *11*, 504.

STAR★METHODS

KEY RESOURCES TABLE

REAGENT or RESOURCE	SOURCE	IDENTIFIER
Deposited data		
The data necessary to reproduce our analysis is publicly available at Mendeley Data.	Mendeley Data	https://doi.org/10.17632/r4r8j6mswx.1
Software and algorithms		
The source code used for this analysis is publicly available at Mendeley Data.	Mendeley Data	https://doi.org/10.17632/r4r8j6mswx.1

RESOURCE AVAILABILITY

Lead contact

A.R. (andrey.rzhetsky@uchicago.edu).

Materials availability

This study did not generate new experimental materials.

Data and code availability

- All data reported in this paper will be shared by the lead contact upon request.
- All original code has been deposited at Mendeley Data and is publicly available as of the date of publication. The DOI is listed in the key resources table.
- Any additional information required to reanalyze the data reported in this work paper is available from the lead contact upon request.

EXPERIMENTAL MODEL AND SUBJECT DETAILS

This study did not use any experimental models.

METHOD DETAILS

In this study, we focused on dissecting the etiology of ten major neuropsychiatric disorders, documented for 138 thousand US families, containing nearly half a million unique individuals. We introduced a collection of increasing-complexity Bayesian models, the simplest of which – traditional mixed-effect generalized linear regression models – contains only fixed-effect demographic and environmental data plus random-effect genetic and environmental factors. The new, more complicated mixed-effect regression models incorporate a series of additional random effects accounting for interactions between genetic and environmental factors. For ten representative neuropsychiatric diseases (most frequently diagnosed in the US), we then estimated how much of the phenotypic variation came from genetic factors, different environmental factors, or interactions between them, using the insurance claim data from 138 thousand US families. Our results suggest that gene-environment interactions account for substantial common neuropsychiatric disorder risk variability.

Data and study population

Our main data resource is the IBM Health MarketScan dataset,[29] which records the health histories of over 150 million unique patients across the US from 2003 to the present. The data also documents both the kinship and county-level place of residence for patients. We chose to analyze individuals who consistently lived in the same geographical location and were enrolled in our data for at least six years, so that we could assume they had been exposed to the same environment for some time. From this filtered population, we selected 138 thousand families (404 thousand individuals), including parents and their above-16-year-old children.

We used a comprehensive collection of raw environmental measurements, deriving from them environmental quality indices (EQIs) developed by the US Environmental Protection Agency (EPA) to quantify the fixed environmental factor in our mixed-effects models.[30,31] For every county, the EQI gave a numerical quality estimation for five domains: air, water, land, sociodemographic, and built environment. It is worth noting that each domain index already summarized many relevant variables, and lower scores

suggest better environmental quality in general. For the air, water, and land domain, the EPA EQIs represent the overall quality considering numerous pollutants and contaminants. The sociodemographic domain represents environmental quality related to income, education, employment, crime, and other socioeconomic elements, while the build-environment domain summarizes factors that are primarily associated with housing quality, proximity to roads, and the intensity of traffic on these roads.

Modeling

We employed a mixed-effects generalized linear regression modeling methodology, using logit-link for binary disease outcome. The fixed-effects included basic demographic factors (such as sex and age groups) and we split the environmental quality indices by categories: air, water, land, sociodemographic, and built-environmental (e.g., housing and highway safety). For the random effects, we designed specific relationship matrices which determined correlation structures across individuals. For example, we specified genetic random effects with a multivariate normal distribution using the genetic relationship matrix (GRM), which indicated the relatedness between individuals. Similarly, environmental random effects shared by family members, couples, or siblings also followed distributions controlled by corresponding relationship matrices (see the Models section of STAR Methods). In addition, we considered the geographic random effects modelled by a Gaussian process (GP). Specifically, the GP's kernel defined the strength of the correlation between two patients' geographic random effects according to the distance between them. We assumed people living closely would share similar environments and, thus, highly-correlated geographic random effects (see the Models section of STAR Methods).

We also considered the interaction between genetic and environmental random effects. By forwardly adding variables, we constructed a group of models from the simple to the more complex. Table 1 summarizes what additive effects were considered in each (named) model. We categorized the models into two forward selection traces: Linear model 0 (LM0) $\vec{x}$ Linear model 1 (LM1) $\vec{x}$ Interaction model 1 (IM1) and Linear model 2 (LM2) $\vec{x}$ Interaction model 2 (IM2). Within each trace, the later models encompassed all the preceding models' variables. The forward selection traces allowed us to determine whether models with additional variables could better describe the data by comparing the information criterion estimates.

Finally, it was possible for us to define statistics that quantified how much outcome variation (logit-probability of disease) could be explained by these additive random effects. For example, heritability (h^2) corresponds to the genetic random effect. In Table 1, we show the statistics p^2, h^2, f^2, c^2, s^2, e^2, hf^2, hc^2, hs^2, and he^2, which represent how much outcome variation can be attributed to the geographic factors, genetic factors, familial environments, couple-shared environments, sibling-shared environments, individually-independent environments, interactions between genetics and familial environments, interactions between genetics and couple-shared environments, interactions between genetics and sibling-shared environments, and interactions between genetics and individual environments, correspondingly. Note that the individually independent environment effects E may include personal experiences in school or the workplace that are not shared with family members, and it could also encompass noises in the clinical assessment or other errors. Please refer to the Models section of STAR Methods for the model definition details.

QUANTIFICATION AND STATISTICAL ANALYSIS

We used a Bayesian procedure to infer parameters of the mixed-effects model. Details about the statistical analysis and software used can be found in Supplemental Information. We conducted statistical simulations to assess whether the inference under the model can untangle effects of genetics, environmental factors, and gene-environment interactions from appropriately simulated data. Details about the statistical simulations can be found in Supplemental Information. This study did not use any methods to determine whether the data met assumptions of the statistical approach.

Models

Linear model 0 (LM0)

For a disease outcome, we assumed that the probability of presenting this disease is p. The logit-probability, defined as $l = \log\left(\frac{p}{1-p}\right)$, could be expressed in an additive mixed-effects model:

$$l = X\beta + f_q(\mathbf{q}) + G + E, \quad \text{(Equation 1)}$$

where X is the design matrix of the demographic fixed effects, including the effects of age and sex. For the fixed effect controlled by environmental quality indices $\mathbf{q} = (q_1, q_2, \ldots, q_m)$, we assumed a polynomial model:

$$f_q(\mathbf{q}) = f_q^{(1)}(q_1) + f_q^{(2)}(q_2) + \ldots + f_q^{(m)}(q_m). \quad \text{(Equation 2)}$$

Our model includes five ($m = 5$) different types of environmental qualities quantified by summary indices: air, water, land, sociodemographic, and build-environment domains. We set the degree of the polynomial function f_q and its components $f_q^{(1)} \ldots f_q^{(m)}$ to be three (cubic).

G is the genetic effect contributing to the phenotype, and E is the individually-independent environmental effect. For an individual group, their genetic effects were associated by the genetic relationship matrix (GRM, Σ_G). As an example, the GRM for a family of two parents and one child should be close to

$$\Sigma_G = \begin{bmatrix} 1.0 & 0.0 & 0.5 \\ 0.0 & 1.0 & 0.5 \\ 0.5 & 0.5 & 1.0 \end{bmatrix}, \quad \text{(Equation 3)}$$

where the first two rows and columns represent the parents, and the last row and column represents the child. Then, we assumed the genetic effects followed a multivariate normal distribution:

$$G \sim \mathrm{MvNormal}\left(\mathrm{mean} = 0,\ \mathrm{cov} = {\sigma_G}^2 \Sigma_G\right). \quad \text{(Equation 4)}$$

The individually-independent environmental effects also followed a multivariate, normal distribution with its covariance equal to a multiple of the identity matrix I:

$$E \sim \mathrm{MvNormal}\left(\mathrm{mean} = 0, \mathrm{cov} = {\sigma_E}^2 I\right). \quad \text{(Equation 5)}$$

In these expressions, ${\sigma_G}^2$ and ${\sigma_E}^2$ are the constants we wanted to find. For a population, the variance-covariance of l is

$$\mathrm{Var}(l) = {\sigma_G}^2 \Sigma_G + {\sigma_E}^2 I. \quad \text{(Equation 6)}$$

Finally, we defined the heritability h^2 and the independent environmental factor e^2 as

$$h^2 = \frac{{\sigma_G}^2}{{\sigma_G}^2 + {\sigma_E}^2}, \quad \text{(Equation 7.1)}$$

$$e^2 = \frac{{\sigma_E}^2}{{\sigma_G}^2 + {\sigma_E}^2}. \quad \text{(Equation 7.2)}$$

Linear model 1 (LM1)

Besides the effects contributed by the basic demographic information (sex and age) and environmental quality, we acknowledged that geographic position might also play a part in disease etiology.

Given a patient's dwelling's latitude and longitude (coordinates) $\mathbf{x} = (x_1, x_2)$, the random effect, as a part of the logit-probability l, is $f_p(\mathbf{x})$, which follows a Gaussian process (GP):

$$f_p(\mathbf{x}) \sim \mathcal{GP}(\mathrm{mean} = 0, \mathrm{cov} = k(\mathbf{x}, \mathbf{x}')). \quad \text{(Equation 8)}$$

The Gaussian process model constrains the distribution of $f_p(\mathbf{x})$, so that the joint distribution of two data points $f_p(\mathbf{x})$ and $f_p(\mathbf{x}')$ is a multivariate normal:

$$\begin{bmatrix} f_p(\mathbf{x}) \\ f_p(\mathbf{x}') \end{bmatrix} \sim \mathrm{MvNormal}\left(\mathrm{mean} = \begin{bmatrix} 0 \\ 0 \end{bmatrix}, \mathrm{cov} = \begin{bmatrix} k(\mathbf{x}, \mathbf{x}) & k(\mathbf{x}, \mathbf{x}') \\ k(\mathbf{x}, \mathbf{x}') & k(\mathbf{x}', \mathbf{x}') \end{bmatrix}\right). \quad \text{(Equation 9)}$$

Therefore, if we chose the kernel function $k(\cdot,\cdot)$ appropriately, we can fit a function $f_p(\mathbf{x})$ that created two random effects $f_p(\mathbf{x})$ and $f_p(\mathbf{x}')$, which would be more similar for coordinates $\mathbf{x}$ and $\mathbf{x}'$ close to each other.

Here, we used the exponentiated quadratic kernel function, which is commonly used in geo-statistics and applications describing quantities distributed in a smooth metric space:

$$k(\mathbf{x}, \mathbf{x}') = {\sigma_P}^2 \cdot \exp\left(-\frac{\mathbf{x} - \mathbf{x}'^2}{2\ell^2}\right), \quad \text{(Equation 10)}$$

where ${\sigma_P}^2$ and ℓ were the scale parameters, we fit through the Markov chain Monte Carlo (MCMC) process.

In all, as a forward addition to the simplest linear model 0 (LM0), we expressed the logit-probability of a disease presenting for an individual as:

$$l = \beta_0 + \beta_{\mathrm{sex}} \cdot \mathrm{Sex} + \beta_{\mathrm{age}} \cdot \mathrm{Age} + f_q(\mathbf{q}) + f_p(\mathbf{x}) + G + E = X\beta + f_q(\mathbf{q}) + f_p(\mathbf{x}) + G + E. \quad \text{(Equation 11)}$$

The variance-covariance of l for a group of individuals was:

$$\mathrm{Var}(l) = {\sigma_P}^2 K_P + {\sigma_G}^2 \Sigma_G + {\sigma_E}^2 I, \quad \text{(Equation 12.1)}$$

where ${\sigma_P}^2 K_P$ was the covariance matrix associating individuals based on their geographic locations, and the row m, column n element of the K_p matrix in Expression (12.1) is defined as follows:

$$K_p(m, n) = \exp\left(-\frac{\mathbf{x}_m - \mathbf{x}_n}{2\ell^2}\right). \quad \text{(Equation 12.2)}$$

This can be derived from the kernel function, Expression (10), as we did in Expression (9).

Consequently, the geographic position factor p^2, which quantifies how much variation can be explained by one's coordinates, the heritability h^2, and the independent environmental factor e^2 were

$$p^2 = \frac{{\sigma_P}^2}{{\sigma_P}^2 + {\sigma_G}^2 + {\sigma_E}^2}, \quad \text{(Equation 13.1)}$$

$$h^2 = \frac{{\sigma_G}^2}{{\sigma_P}^2 + {\sigma_G}^2 + {\sigma_E}^2}, \quad \text{(Equation 13.2)}$$

$$e^2 = \frac{{\sigma_E}^2}{{\sigma_P}^2 + {\sigma_G}^2 + {\sigma_E}^2}. \quad \text{(Equation 13.3)}$$

Interaction Model 1 (IM1)

We then considered the interaction between the genetic effect and the environmental effect. The logit-probability of having a disease can be expressed as

$$l = X\beta + f_q(\mathbf{q}) + f_p(\mathbf{x}) + G + E + k_{GE} \cdot G \cdot E, \quad \text{(Equation 14)}$$

The scale factor k_{GE} determines how much effect the interaction contributes to the phenotype. G and E were the same genetic and environmental effects as they were given in the linear models 0 and 1.

The variance-covariance of l was

$$\mathrm{Var}(l) = {\sigma_P}^2 K_P + {\sigma_G}^2 \Sigma_G + {\sigma_E}^2 I + {k_{GE}}^2 {\sigma_G}^2 {\sigma_E}^2 \Sigma_G \odot I, \quad \text{(Equation 15)}$$

Note that the above expression, demonstrates the method by which we fit the model specified in Expression (14), not (15). Expression (15) functioned as an intermediate step for calculating the total variance based on the estimates of ${\sigma_p}^2$, ${\sigma_G}^2$, ${\sigma_E}^2$, etc. $\Sigma_G \odot I$ is equal to Σ_G, but this did not affect the identifiability of the model defined in Expression (14) considering that random vector G is certainly not equal to the random vector multiplication $G \times E$ (as the elements in the random vector E are not all ones).

We assumed that the genetic effect and the environmental effects are statistically independent. The operator $\odot$ represents the elementwise (Hadamard) product. The geographic position factor p^2, the heritability h^2, and the independent environmental factor e^2 are

$$p^2 = \frac{{\sigma_P}^2}{{\sigma_P}^2 + {\sigma_G}^2 + {\sigma_E}^2 + {k_{GE}}^2 {\sigma_G}^2 {\sigma_E}^2}, \quad \text{(Equation 16.1)}$$

$$h^2 = \frac{{\sigma_G}^2}{{\sigma_P}^2 + {\sigma_G}^2 + {\sigma_E}^2 + {k_{GE}}^2 {\sigma_G}^2 {\sigma_E}^2}, \quad \text{(Equation 16.2)}$$

$$e^2 = \frac{{\sigma_E}^2}{{\sigma_P}^2 + {\sigma_G}^2 + {\sigma_E}^2 + {k_{GE}}^2 {\sigma_G}^2 {\sigma_E}^2}. \quad \text{(Equation 16.3)}$$

Furthermore, interactions between genetics and the environment can also explain the variance of the logit-probability. We defined a new interaction factor

$$he^2 = \frac{{k_{GE}}^2 {\sigma_G}^2 {\sigma_E}^2}{{\sigma_P}^2 + {\sigma_G}^2 + {\sigma_E}^2 + {k_{GE}}^2 {\sigma_G}^2 {\sigma_E}^2}, \quad \text{(Equation 17)}$$

Linear Model 2 (LM2)

The linear models 0 and 1 incorporate the individually-independent environmental effect E only. However, because family members, couples, and siblings may have shared similar behaviors and milieus, we also included other types of environmental effects. Here, the linear model 2 consider the family effect F, the couple effect C, and the sibling effect S in addition to the individually-independent environmental effect E:

$$l = X\beta + f_q(\mathbf{q}) + f_p(\mathbf{x}) + G + F + C + S + E. \quad \text{(Equation 18)}$$

These additional environmental effects were assumed to follow multivariate normal distributions:

$$F \sim \mathrm{MvNormal}\left(\text{mean} = 0, \text{cov} = {\sigma_F}^2 \Sigma_F\right), \quad \text{(Equation 19.1)}$$

CellPress
OPEN ACCESS

$$C \sim \text{MvNormal}\left(\text{mean} = 0, \text{cov} = {\sigma_C}^2 \Sigma_C\right), \quad \text{(Equation 19.2)}$$

$$S \sim \text{MvNormal}\left(\text{mean} = 0, \text{cov} = {\sigma_S}^2 \Sigma_S\right). \quad \text{(Equation 19.3)}$$

We determined the relationship matrices Σ_F, Σ_C, and Σ_S by how much of the kinship-specific environment they shared on average. The relationship matrices for a family of two parents (shown in the first two rows and columns) and two children (shown in the last two rows and columns) are:

$$\Sigma_F = \begin{bmatrix} 1.0 & 1.0 & 1.0 & 1.0 \\ 1.0 & 1.0 & 1.0 & 1.0 \\ 1.0 & 1.0 & 1.0 & 1.0 \\ 1.0 & 1.0 & 1.0 & 1.0 \end{bmatrix}, \quad \text{(Equation 20.1)}$$

$$\Sigma_C = \begin{bmatrix} 1.0 & 1.0 & 0.0 & 0.0 \\ 1.0 & 1.0 & 0.0 & 0.0 \\ 0.0 & 0.0 & 1.0 & 0.0 \\ 0.0 & 0.0 & 0.0 & 1.0 \end{bmatrix}, \quad \text{(Equation 20.2)}$$

$$\Sigma_S = \begin{bmatrix} 1.0 & 0.0 & 0.0 & 0.0 \\ 0.0 & 1.0 & 0.0 & 0.0 \\ 0.0 & 0.0 & 1.0 & 1.0 \\ 0.0 & 0.0 & 1.0 & 1.0 \end{bmatrix}. \quad \text{(Equation 20.3)}$$

The variance-covariance of l can then be estimated:

$$\text{Var}(l) = {\sigma_P}^2 K_P + {\sigma_G}^2 \Sigma_G + {\sigma_F}^2 \Sigma_F + {\sigma_C}^2 \Sigma_C + {\sigma_S}^2 \Sigma_S + {\sigma_E}^2 I. \quad \text{(Equation 21)}$$

We defined the heritability h^2 and other statistics specifying environmental effects as:

$$p^2 = \frac{{\sigma_P}^2}{{\sigma_P}^2 + {\sigma_G}^2 + {\sigma_F}^2 + {\sigma_C}^2 + {\sigma_S}^2 + {\sigma_E}^2}, \quad \text{(Equation 22.1)}$$

$$h^2 = \frac{{\sigma_G}^2}{{\sigma_P}^2 + {\sigma_G}^2 + {\sigma_F}^2 + {\sigma_C}^2 + {\sigma_S}^2 + {\sigma_E}^2}, \quad \text{(Equation 22.2)}$$

$$f^2 = \frac{{\sigma_F}^2}{{\sigma_P}^2 + {\sigma_G}^2 + {\sigma_F}^2 + {\sigma_C}^2 + {\sigma_S}^2 + {\sigma_E}^2}, \quad \text{(Equation 22.3)}$$

$$c^2 = \frac{{\sigma_C}^2}{{\sigma_P}^2 + {\sigma_G}^2 + {\sigma_F}^2 + {\sigma_C}^2 + {\sigma_S}^2 + {\sigma_E}^2}, \quad \text{(Equation 22.4)}$$

$$s^2 = \frac{{\sigma_S}^2}{{\sigma_P}^2 + {\sigma_G}^2 + {\sigma_F}^2 + {\sigma_C}^2 + {\sigma_S}^2 + {\sigma_E}^2}, \quad \text{(Equation 22.5)}$$

$$e^2 = \frac{{\sigma_E}^2}{{\sigma_P}^2 + {\sigma_G}^2 + {\sigma_F}^2 + {\sigma_C}^2 + {\sigma_S}^2 + {\sigma_E}^2}. \quad \text{(Equation 22.6)}$$

Interaction model 2 (IM2)

Similar to our process in the experimental models 1 and 2, we added interaction terms to the above-defined linear model 2. Thus, in this model, the logit-probability of having a disease is:

$$I = X\beta + f_q(\mathbf{q}) + f_p(\mathbf{x}) + G + F + C + S + E + k_{GF}\cdot G\cdot F + k_{GC}\cdot G\cdot C + k_{GS}\cdot G\cdot S + k_{GE}\cdot G\cdot E. \quad \text{(Equation 23)}$$

The variance-covariance of I is:

$$\begin{aligned}\mathrm{Var}(I) = {} & \sigma_P{}^2 K_P + \sigma_G{}^2\Sigma_G + \sigma_F{}^2\Sigma_F + \sigma_C{}^2\Sigma_C + \sigma_S{}^2\Sigma_S + \sigma_E{}^2 I + k_{GF}{}^2\,\sigma_G{}^2\,\sigma_F{}^2\,\Sigma_G\odot\Sigma_F + k_{GC}{}^2\,\sigma_G{}^2\,\sigma_C{}^2\,\Sigma_G\odot\Sigma_C \\ & + k_{GS}{}^2\,\sigma_G{}^2\,\sigma_S{}^2\,\Sigma_G\odot\Sigma_S + k_{GE}{}^2\,\sigma_G{}^2\,\sigma_E{}^2\,\Sigma_G\odot I.\end{aligned} \quad \text{(Equation 24)}$$

We defined the heritability h^2 and statistics specifying the environmental effects (geographic position: p^2, family: f^2, couple: c^2, sibling: s^2, and independent: e^2) as we did in Expressions 16 and 22. Likewise, statistics quantifying the genetic interactions (genetics-family: hf^2, genetics-couple: hc^2, genetics-sibling: hs^2, and genetics-individual-environment: he^2) were given according to their partitions in Var(I), similar to the Expression 17.

Bayesian inference

The models specified in Expressions (1), (11), (14), (18), and (23) are nonlinear mixed-effects models, which we can represent in a general form:

$$I = f(X\beta,\ k_1Z_1,\ldots,\ k_nZ_n), \quad \text{(Equation 25)}$$

where $X\beta$ are fixed effects, and $Z_1\ldots Z_n$ are random effects controlled by certain association matrices Σ_Z – such as the individually-independent environmental effects as given in Expression (5) – and the genetic effect G constrained by the pedigree:

$$Z_n \sim \mathrm{MvNormal}\left(\mathrm{mean} = 0, \mathrm{cov} = \sigma_{Z_n}{}^2\Sigma_Z\right). \quad \text{(Equation 26)}$$

k_n is the scale factor for the random effects Z_n. Our target was to estimate not only the coefficients β and $k = \{k_1,\ldots,k_n\}$ but also the variances $\sigma^2 = \{\sigma_{Z_1}{}^2\ldots\sigma_{Z_n}{}^2\}$. To accomplish this, we started by considering the likelihood function

$$L(\beta,k,\sigma^2,Z|y) = \Pr(y|I = f(X\beta,\ k_1Z_1,\ldots,\ k_nZ_n)), \quad \text{(Equation 27)}$$

where y is the binary disease outcome linked to the logit-probability $I = \log\left(\frac{p}{1-p}\right)$, and $Z = \{Z_1\ldots Z_n\}$ is a collection representing all random effects – similar to the collections k and σ^2.

However, this likelihood was not computable because of the unobserved random effects Z. To find the maximum likelihood (ML) estimates for β, k, and σ^2, we had to integrate out the random term Z:

$$L(\beta,k,\sigma^2|y) = \int \Pr(y|I = f(X\beta,\ k_1Z_1,\ldots,\ k_nZ_n))\Pr(Z|\sigma^2)\mathrm{d}Z. \quad \text{(Equation 28)}$$

We were then free to apply suitable methods, such as stochastic gradient decant (SGD) or the expectation-maximization algorithm (EM) to approximate the maximum likelihood estimates – with one caveat: The ML approaches are known to underestimate the variances.σ[32,33] There exist modifications of ML methods, for example, restricted maximum likelihood (REML), in which both the random term Z and the fixed effects coefficient β would be integrated out in order to better estimate the variances.[34] But the REML also has its limitations; because of the integration of the fixed components, comparison would not be sensible between models of different fixed effects design.[35]

We chose to use a fully Bayesian framework to avoid the drawbacks of ML and other related Frequentist methods. Solving the model with a Bayesian's view provided several advantages towards solving our problem. First, for any statistical model, the Bayesian method follows an intuitive, easy-to-construct flow – model and prior specification first, and all the inferences would be automated on the computer's side. The model-centered, uniform flow allowed us to focus our attention on exploring various models (LM0 to IM2) and their biological interpretations – with no liability to solve a novel nonlinear model. Additionally, Bayesian methods allowed us to quantify the statistical uncertainty in a natural manner because the inference would be done in a sampling process (MCMC). In a fully Bayesian framework, mean, credible intervals, or other distribution statistics are computable upon obtaining the samplings. By contrast, the ML and other related Frequentist methods do not provide an integrated method for estimate inferences and their uncertainties – the ML estimations may involve first-order approaches like SGD, while approximating the confidence intervals involved typically require a separated calculation of the likelihood's higher-order derivative.[36,37] Bayesian methods also offer further benefits such as model comparisons, especially when working with high-dimensional models (WAIC and PSIS-LOO), and versatile regularization approaches through shrinkage and moldable priors.[38,39]

Priors

To find the posterior distribution of a parameter θ under a Bayesian framework, we first needed to specify the prior according to the Bayes theorem $P(\theta|\mathrm{Data}) \propto P(\theta) \times P(\mathrm{Data}|\theta) = \mathrm{Prior} \times \mathrm{Likelihood}$. We avoided overfitting through implementing Bayesian shrinkage priors. The simplest example of shrinkage priors is Laplace distribution, which puts more weight in samplings close to zero and therefore rewards underfit estimates. We used the horseshoe shrinkage prior[40] for the fixed-effects parameters (like the demographic predictor parameter β in the Expression 1 and the polynomial function parameter in Expression 2), so we imposed sparsity and regularization to avoid overly complicated models. For parameters restricted to be positive, such as the variance scale factor $\sigma_G{}^2$ and

$\sigma_E{}^2$ in Expression 6, we used the zero-avoiding Gamma prior, as recommended by Chung et al.[41] and the Stan prior choice recommendations.[42]

Sampling

After specifying the hierarchical models with priors and likelihood functions, Bayesian inference methods rely on a sampling algorithm (sampler) to draw from the posterior distribution and approximate the posterior efficiently. In the present study, we used Hoffman and Gelman's No-U-Turn sampler (NUTS)[24] designed for high-dimensional, ill-shaped target distributions. The No-U-Turn sampler tunes the step size automatically and directs sampling by looking to the gradient information. Compared to vanilla Markov chain Monte Carlo methods, the No-U-Turn sampler[24] more quickly depicts complex posterior distributions. We initialized the sampling process using automatic differentiation variational inference (ADVI),[43] providing the MCMC with an optimized start similar to the Frequentist's maximum likelihood methods.[44] We then sampled four times independently using NUTS to estimate all the variables. The mean estimates were calculated by combining all four independent samplings to avoid possible sampling instability.

Statistical simulations of inference under the assumed model

Compared to prior research in heritability estimation, our model incorporates additional effects of gene-environment interactions. The identifiability and performance might appear questionable for estimation under the new model. Therefore, we conducted statistical simulations to assess whether the inference under the model can untangle effects of genetics, environmental factors, and gene-environment interactions from appropriately simulated data, see Table S1.

We considered a simplified interaction model. The logit-probability of having a disease can be expressed as

$$l = X\beta + f_q(\mathbf{q}) + G + E + k_{GE}\cdot G\cdot E, \qquad \text{(Equation 29)}$$

where X is the design matrix of the demographic fixed effects, including the effects of age and sex. $f_q(\mathbf{q})$ is a cubic polynomial function modeling the fixed effects of environmental quality indices, including air, water, land, sociodemographic, and build-environment domains. G is the random effect of genetic factors, E is the random effects of environmental factors, and $k_{GE}\cdot G\cdot E$ quantifies the influence of gene-environment interactions.

We assigned five sets of genetic variance scale $\sigma_G{}^2$ and environmental variance scale $\sigma_E{}^2$: $\sigma_G{}^2 = 1, \sigma_E{}^2 = 5$; $\sigma_G{}^2 = 2, \sigma_E{}^2 = 4$; $\sigma_G{}^2 = 3, \sigma_E{}^2 = 3$; $\sigma_G{}^2 = 4, \sigma_E{}^2 = 2$; and $\sigma_G{}^2 = 5, \sigma_E{}^2 = 1$. The scale factor k_{GE} is set to be $\frac{1}{3}$, and the gene-environment interactions can be determined accordingly. These five experimental sets cover diverse phenotypic conditions regarding the genetic and environmental influences, ranging from dominantly environmental to dominantly genetic traits. The true h^2, e^2, and he^2 can be calculated using the following expressions

$$h^2 = \frac{\sigma_G{}^2}{\sigma_G{}^2 + \sigma_E{}^2 + k_{GE}{}^2\,\sigma_G{}^2\,\sigma_E{}^2}, \qquad \text{(Equation 30)}$$

$$e^2 = \frac{\sigma_E{}^2}{\sigma_G{}^2 + \sigma_E{}^2 + k_{GE}{}^2\,\sigma_G{}^2\,\sigma_E{}^2}. \qquad \text{(Equation 31)}$$

$$he^2 = \frac{k_{GE}{}^2\,\sigma_G{}^2\,\sigma_E{}^2}{\sigma_G{}^2 + \sigma_E{}^2 + k_{GE}{}^2\,\sigma_G{}^2\,\sigma_E{}^2}, \qquad \text{(Equation 32)}$$

For each experimental set, we sampled 1,000 families from our data, and generated corresponding binary phenotype with the logit-probability defined in Expression 29. Given the simulated phenotypic data, family relationship information, and the model, we then conducted the Bayesian inference using the method we descried in the *Bayesian Inference* section of the supplementary information.

We repeated this sampling and inference process 100 times for each experimental set and computed the corresponding 95 percent credible intervals for h^2, e^2, and he^2. By examining if the pre-set, true h^2, e^2, and he^2 fell in the credible intervals, we can estimate the coverage probability

$$\text{Coverage probability} = \Pr(C_{lo} \le s \le C_{up}), \qquad \text{(Equation 33)}$$

where s is the true value of a statistic (h^2, e^2, or he^2), and (C_{lo}, C_{up}) is the corresponding credible interval for s, which we estimated 100 times independently with different family samples.

Cell Reports Medicine, Volume *3*

Supplemental information

Gene-environment interactions explain a substantial portion of variability of common neuropsychiatric disorders

Hanxin Zhang, Atif Khan, and Andrey Rzhetsky

Gene-Environment Interactions Explain a Substantial Portion of Variability of Common Neuropsychiatric Disorders: Supplementary Figures and Table

Hanxin Zhang[1, 2], Atif Khan[2], and Andrey Rzhetsky[1, 2, 3, 4 *]

[1]Committee on Genetics, Genomics and Systems Biology, The University of Chicago, Chicago, IL, 60637, US.

[2]Department of Medicine, and Institute of Genomics and Systems Biology, The University of Chicago, Chicago, IL, 60637, US.

[3]Department of Human Genetics and Committee on Quantitative Methods in Social, Behavioral, and Health Sciences, The University of Chicago, Chicago, IL, 60637, US.

[4]Lead contact

[*]Correspondence to: andrey.rzhetsky@uchicago.edu

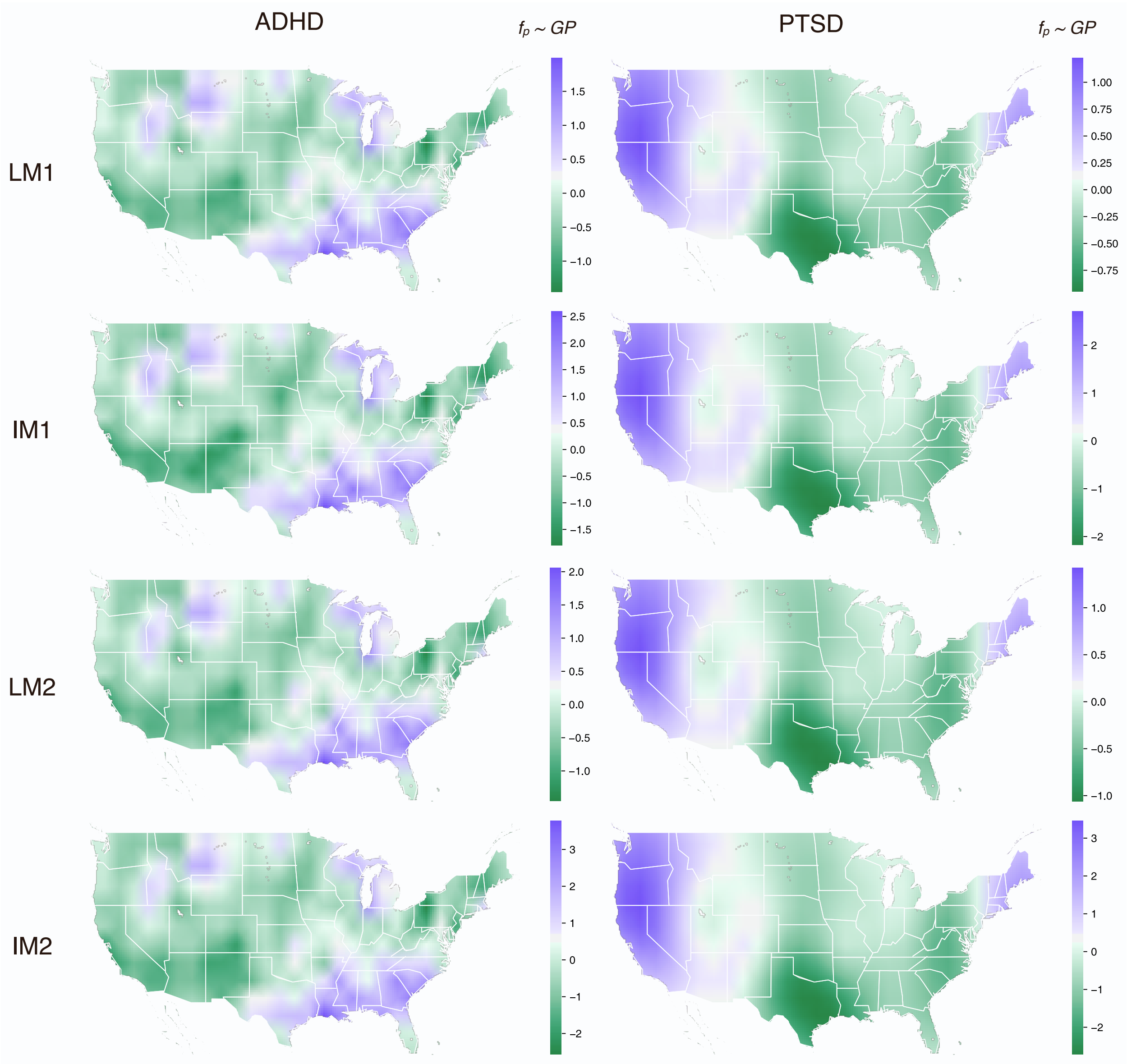

Figure S1. The mean estimates of the geographic random effects for ADHD and PTSD, related to Figure 1.

These figure plots show the posterior mean estimates of the geographic random effects for ADHD and PTSD ($f_p(\mathbf{x})$ (see Expression (8) in the *Models* section of the *Supplementary Information*). We modeled the geographic random effects $f_p(\mathbf{x})$ using a Gaussian process assuming that adjacent geographical locations have close-value random effects (assumption of smoothness). For both ADHD and PTSD, all models across the complexity spectrum (LM1, IM1, LM2, and IM2; note that LM0 does not incorporate the geographic random effect term) give nearly identical patterns of $f_p(\mathbf{x})$ estimates across the continental US. We did include residents of Hawaii and Alaska in our estimation process, but the results are not shown here. We omitted these results because the discontinuity between the geographic locations disobeys the Gaussian process's presumptions. The Gaussian process's poor extrapolation power also makes it difficult to estimate the random effects related to outlying states such as Alaska and Hawaii. Our data does not record any residents of other non-continental US islands.

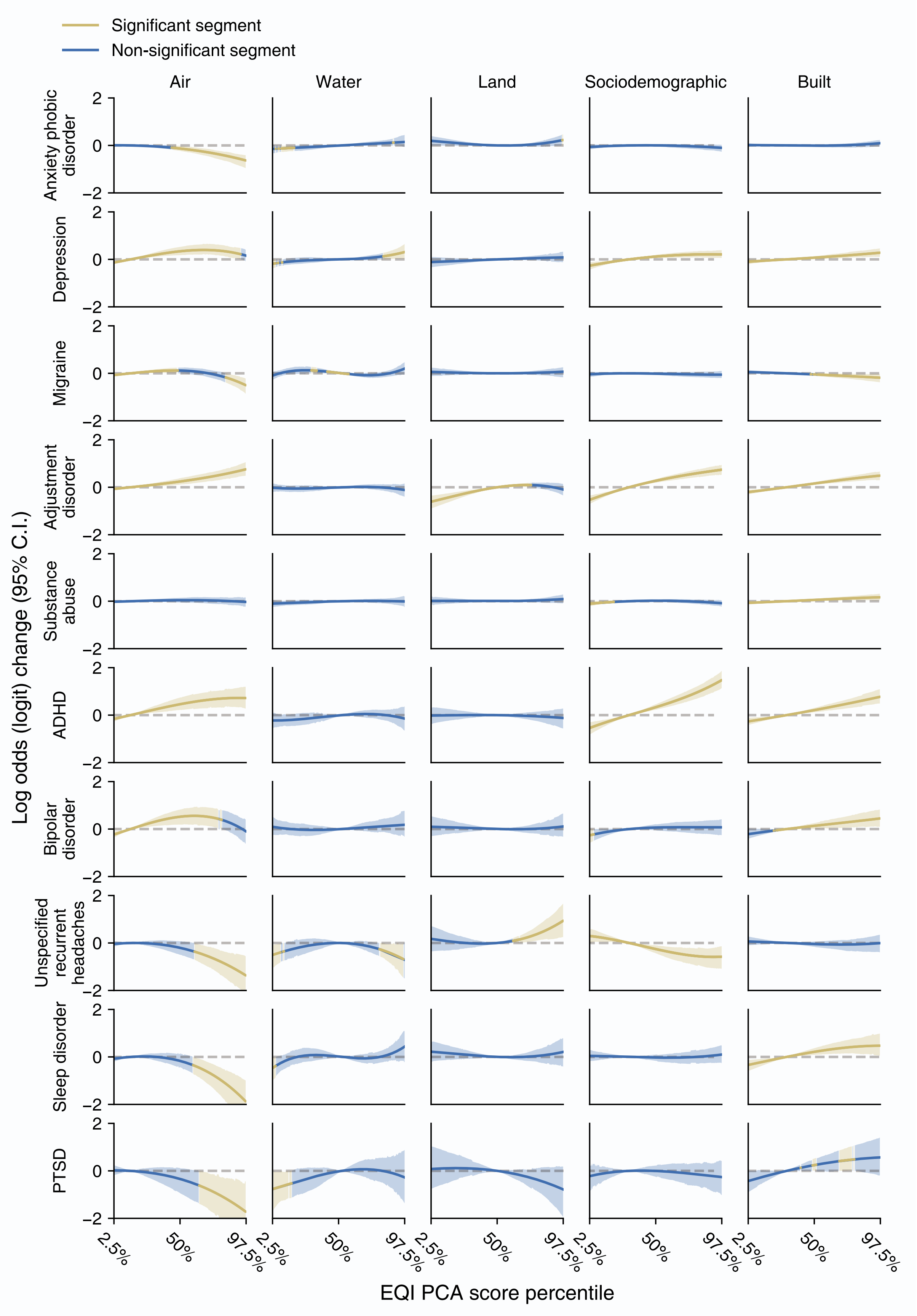

Significant segment
Non-significant segment
Air
Water
Land
Sociodemographic
Built
Anxiety phobic disorder
Depression
Migraine
Adjustment disorder
Substance abuse
ADHD
Bipolar disorder
Unspecified recurrent headaches
Sleep disorder
PTSD
Log odds (logit) change (95% C.I.)
2
0
−2
2.5%
50%
97.5%
EQI PCA score percentile

Figure S2. The nonlinear effects estimated for the five EQI domains (air, water, land, sociodemographic, and built environment) given by each disease's WAIC-best model, related to Figure 2.

Each EQI domain contains multiple relevant variables. We employed a PCA procedure to summarize and standardize domain-specific variables into one-dimensional PCA scores. According to the publisher (EPA), higher scores generally reflect worse environmental quality. We converted these PCA scores into percentiles based on the score distribution covering the whole population. We used the 2.5th to 97.5th percentiles as the x-axis limits of the plots. In the line plots, the blue segments indicate EQI regions that do not change the log odds significantly (statistically equal to zero, 95 percent credible intervals). The olive segments represent those EQI regions that significantly alter the disease log odds (statistically non-zero regions, 95 percent credible intervals).

Supplementary Information Tables

Table S1. The simulation result of the five experiment sets (see Section *Statistical Simulations of Inference Under the Assumed Model* in the *STAR Methods*).

Experiment set 1: ${\sigma_G}^2 = 1, {\sigma_E}^2 = 5$

**Statistics**	h^2	e^2	he^2
**Coverage probability estimate**	0.98	0.78	0.92

Experiment set 2: ${\sigma_G}^2 = 2, {\sigma_E}^2 = 4$

**Statistics**	h^2	e^2	he^2
**Coverage probability estimate**	0.90	0.86	0.88

Experiment set 3: ${\sigma_G}^2 = 3, {\sigma_E}^2 = 3$

**Statistics**	h^2	e^2	he^2
**Coverage probability estimate**	0.89	0.97	0.94

Experiment set 4: ${\sigma_G}^2 = 4, {\sigma_E}^2 = 2$

**Statistics**	h^2	e^2	he^2
**Coverage probability estimate**	0.86	0.98	0.93

Experiment set 5: ${\sigma_G}^2 = 5, {\sigma_E}^2 = 1$

**Statistics**	h^2	e^2	he^2
**Coverage probability estimate**	0.83	0.89	0.89

Average coverage probability estimate = 0.90
